# One-Dimensional La_0.2_Sr_0.8_Cu_0.4_Co_0.6_O_3−δ_ Nanostructures for Efficient Oxygen Evolution Reaction

**DOI:** 10.3390/nano14010064

**Published:** 2023-12-26

**Authors:** Dongshuang Wu, Yidan Chen, Yuelei Bai, Chuncheng Zhu, Mingyi Zhang

**Affiliations:** 1 Key Laboratory for Photonic and Electronic Bandgap Materials, Ministry of Education, School of Physics and Electronic Engineering, Harbin Normal University, Harbin 150025, China; 2 National Key Laboratory of Science and Technology on Advanced Composites in Special Environments, Harbin Institute of Technology, Harbin 150001, China

**Keywords:** perovskite, OER, nanofiber, electrospinning

## Abstract

Producing oxygen and hydrogen via the electrolysis of water has the advantages of a simple operation, high efficiency, and environmental friendliness, making it the most promising hydrogen production method. In this study, La_0.2_Sr_0.8_Cu_0.4_Co_0.6_O_3−δ_ (LSCC) nanofibers were prepared by electrospinning to utilize non-noble perovskite oxides instead of noble metal catalysts for the oxygen evolution reaction, and the performance and electrochemical properties of LSCC nanofibers synthesized at different firing temperatures were evaluated. In an alkaline environment (pH = 14, 6 M KOH), the nanofibers calcined at 650 °C showed an overpotential of 209 mV at a current density of 10 mA cm^−2^ as well as good long-term stability. Therefore, the prepared LSCC-650 NF catalyst shows excellent potential for electrocatalytic oxygen evolution.

## 1. Introduction

Metal-air batteries and fuel cells have been widely investigated to address global environmental and energy issues [1,2,3,4,5]. However, while these energy conversion and storage systems are environmentally friendly and have high energy density, their commercialization has been limited by the slow kinetics of the oxygen evolution reaction (OER) [6,7,8]. The oxygen evolution reaction is a semi-anodic reaction that occurs during water electrolysis. OER involves a four-electron transfer process, which is characterized by its high energy requirements, slow chemical kinetics, and complex mechanisms. The OER process involves a series of sequential reactions in an alkaline/neutral electrolyte: first, OH^−^ or H_2_O adsorbed on an active site is oxidized with an electron to form OH_ad_. Second, the removal of a proton and electron from OH_ad_ forms O_ad_. Finally, O_ad_ is transformed by one of two potential pathways to form a detached O_2_ molecule. In one pathway, two O_ad_ directly combine to form O_2_. In the second pathway, O_ad_ reacts with H_2_O or OH^−^ to form the intermediate product OOH_ad_, which combines with H_2_O or OH^−^ to form O_2_ (Figure S1) [9,10,11]. Numerous research studies have focused on exploring synthetic materials for the preparation of suitable OER catalysts with the goal of improving the performance of water electrolysis electrodes. Two crucial factors that significantly contribute to improved electrode performance are stability and drivability. Within the realm of electrochemistry, catalysts play an indispensable role in facilitating desired chemical reactions at the surface of the electrode. Catalysts have the ability to increase reaction rates, reduce energy barriers, and enhance the efficiency of electrochemical processes. Consequently, the development of catalysts that are both efficient and stable holds immense significance.

Many synthetic materials such as RuO_2_ and IrO_2_ have been evaluated as OER catalysts due to their exceptional performance across various pH environments [12,13,14]. However, noble metal-based catalysts have the disadvantages of resource scarcity and a high cost. Therefore, a growing body of research has focused on the evaluation of a diverse range of non-noble metal oxides as catalysts. In particular, perovskite materials with ABO_3_ structures, spinels with A’B’_2_O_4_ structures, and composite oxide materials with layered structures all exhibit excellent performance as electrocatalysts [15,16,17,18,19,20,21,22,23,24,25,26,27,28]. In addition to metal oxides, metal sulfides composed of sulfur groups, non-oxide catalysts, and metal coordination compounds have also shown excellent promise in the field of electrocatalysis [29,30,31,32,33,34,35,36,37]. These materials offer high catalytic activity, good electrical conductivity, and excellent chemical stability, which are essential factors for improving the performance of electrodes. Transition metal-based oxides have the advantages of a low cost, an easy synthesis, and good environmental protection. In addition, these oxides are good candidates for electrocatalytic OER because they are highly stable in alkaline solutions and have good electrical conductivity. Moreover, because transition metal oxidation states are relatively active and these oxides have relatively free coordination environments, various oxide catalysts can be obtained through rich combinations of transition metals, including oxides with perovskite structures. Thus, metal oxide catalysts exhibit a wide range of properties and performance. Perovskite-based electrocatalysts are considered to be highly promising for OER due to their excellent chemical, physical, and catalytic properties (such as high ionic conductivity and rapid oxidation exchange). Moreover, these materials are environmentally friendly and have low-cost synthesis procedures. For example, La_0.6_Sr_0.4_CoO_3_, LaNiO_3_, and La_1−x_Ce_x_NiO_3_ have been evaluated as OER electrocatalysts [38,39,40].

Conventional strategies such as sol-gel, hydrothermal, co-precipitation, and electrospinning methods can be used to synthesize perovskite oxide nanomaterials [41,42,43,44,45,46,47,48,49,50,51,52]. Compared with other methods, electrospun nanofibers show great potential in OER applications due to their excellent inherent properties and obvious advantages. For instance, electro spun nanofibers have unique physical and chemical properties, including ultra-long length, good electrical conductivity, excellent stability, and good mechanical strength. Moreover, electrospinning can be used to build a very rich pore structure, which can promote rapid mass and electron transfer [53]. The electrocatalytic performance of electrospun nanofibers can be improved by the addition of active substances during the electrospinning process to produce a layered structure [49,54]. Due to the controllability and unique structures of these nanofibers, electrocatalytic activity can be further enhanced by other strategies such as heteroatom doping and surface modification [55,56]. Finally, electrospun nanofibers can be converted into carbon nanofibers (CNFs) or non-carbon nanofibers as well as derivatives rich in nanoparticles by appropriately controlling the atmosphere and annealing temperature. This can also significantly enhance electrocatalytic performance. Because of these advantages, electrospun nanomaterials have been widely evaluated as OER electrocatalysts [57,58,59]. One main focus in the field of OER electrocatalysts is the synthesis of nanomaterials with large specific surface areas. This is because oxygen molecules are mainly adsorbed and deionized on the surface and interface of the catalyst during the OER reaction. Thus, high-surface-area and easily modified electrospun nanofibers show excellent promise as OER electrocatalysts.

In this study, La_0.2_Sr_0.8_Cu_0.4_Co_0.6_O_3−δ_ (LSCC) nanofibers (NFs) with a perovskite structure and large specific surface area were synthesized by electrospinning. The morphology and electrocatalytic performance of the LSCC NFs calcined at different temperatures (500–800 °C) were studied. LSCC-650 NFs, which were obtained at a calcination temperature of 650 °C, show a lower overpotential and Tafel slope than other perovskite materials (Table S1).

## 2. Experimental

### 2.1. Chemical Reagents

Sigma-Aldrich provides polyacrylonitrile (PAN). Cobalt nitrate hexahydrate (Co(NO_3_)_2_·6H_2_O), copper nitrate pentahydrate (Cu(NO_3_)_2_·5H_2_O), strontium nitrate (Sr(NO_3_)_2_), lanthanum nitrate hexahydrate (La(NO_3_)_3_·6H_2_O), N-dimethylformamide (DMF), and potassium hydroxide (KOH) were purchased from Zhiyuan Reagent (Tianjin, China). 2-Methylimidazole were provided from Aladdin (Beijing, China). Analytical grade chemicals that have not been further purified were used in this study.

### 2.2. Synthesis of PAN/LSCC Nanofibers

A 0.2 g amount of 2-methylimidazole and 0.2 g of PAN powder were added to 4 mL DMF. This solution was stirred for 12 h at room temperature using a magnetic stirrer to ensure even mixing. Next, 86.60 mg of lanthanum nitrate, 169.31 mg of strontium nitrate, 174.62 mg of cobalt nitrate, and 96.64 mg of copper nitrate pentahydrate were mixed in a molar ratio of 0.2:0.8:0.4:0.6 and dispersed in the DMF solution. This mixed precursor solution was loaded into a syringe for electrospinning. The electrospinning process can be divided into three main steps: charge generation, charge acceleration, and fiber curing. First, a high voltage is generated by a charge-generating device, such as a high-voltage generator. The high voltage ionizes the molecules in the material, releasing positive and negative charges. The charged material is then ejected through a nozzle while being subjected to an electric field. The positive and negative charges are accelerated in an electric field, forming slender fibers. Finally, these fibers solidify rapidly in the air to form fiber structures with specific properties. After 12 h of high-pressure spraying, we can obtain a thick circular fiber overlap on the receiver. Electrospinning was performed with a working voltage of 6 kV, an acquisition distance of 15 cm, and a humidity of 20–30%.

### 2.3. Synthesis of LSCC Nanofibers

The synthesized PAN/LSCC nanofibers were annealed in the furnace at the temperatures of 500 °C, 550 °C, 600 °C, 650 °C, 700 °C, 750 °C, and 800 °C for 2 h with a heating rate of 1 °C min^−1^ under air atmosphere, respectively. Correspondingly, the obtained products were denoted as LSCC-500 NFs, LSCC-550 NFs, LSCC-600 NFs, LSCC-650 NFs, LSCC-700 NFs, LSCC-750 NFs, and LSCC-800 NFs.

### 2.4. Materials Characterization

The crystal structure of prepared materials was determined by X-ray diffraction (XRD, Rigaku D/max2600, Tokyo, Japan). The atomic structure of catalysts was observed using transmission electron microscopy (TEM, FEI, Tecnai TF20, Tokyo, Japan). The surface chemical properties of the composite were determined by X-ray photoelectron spectroscopy (XPS, Kratos, AXIS SUPRA+, Tokyo, Japan). The morphologies of the samples were observed by scanning electron microscopy (SEM, SU70, Hitachi, Tokyo, Japan). Energy-dispersive X-ray (EDX, QUANTAX, Bruker, Beijing, China) spectroscopy in conjunction with scanning electron microscopy was used to analyze the composition of the samples.

### 2.5. Electrochemical Measurements

Functional electrodes were prepared using 15 mg of LSCC, PVDF, and acetylene black in a mass ratio of 8:1:1. PVDF was used as the binder, while acetylene black was used as the conductor. This mixture was added to a small amount of N-methyl pyrrolidone solvent (NMP), and the resulting slurry was vigorously stirred and mixed, and subsequently ultrasonicated for 2 h to obtain a uniform solution. After ultrasonication, the mixture was evenly coated on carbon paper (1 × 1 cm^2^), which was then dried under a vacuum at 50 °C for 12 h. The resulting sample contained an active catalyst loading of about 4 mg cm^−2^.

Electrochemical tests were performed in a standardized VMP-300 electrochemical apparatus using a three-electrode system. The reaction environment for all electrochemical studies was 6 M KOH. A platinum plate was used as the counter electrode, an Ag/AgCl electrode was used as the reference electrode, and the catalyst sample was used as the working electrode. All experimental results were evaluated in terms of the reversible hydrogen electrode (RHE): E_RHE_ = E_SCE_ + 0.059 pH + 0.196 V (pH = 14), overpotential: *ŋ* = E_RHE_ − 1.23 V. Cyclic voltammetry (CV) was performed for 50 cycles. Good activity and accurate CV curves were obtained with a scanning rate of 50 mV s^−1^. Linear sweep voltammetry (LSV) was performed at a scanning rate of 5 mV s^−1^. The overpotential (*η*), the Tafel slope (b), and current density (j) were evaluated using the equation *η* = b log (*j*) + a. Electrochemical impedance spectroscopy (EIS) was performed at a voltage of 500 mV in the frequency range from 0.01 Hz to 100 kHz. The non-Faraday current regions were evaluated by CV to obtain the electrochemical surface area (ECSA). The double-layer capacitance of each sample was determined by obtaining a CV curve at a special scanning rate. Scanning rates of from 20 to 100 mV s^−1^ with an interval of 20 mV s^−1^ were evaluated in this study. Electrocatalytic stability testing was performed at a current density of 10 mA cm^−2^, and stability was evaluated by chronopotentiometry after 48 h. Cyclic stability was evaluated by conducting 5000 consecutive cyclic tests at 100 mV s^−1^. All potentials were compensated and corrected with 95% IR.

## 3. Results and Discussion

**Scheme 1:**
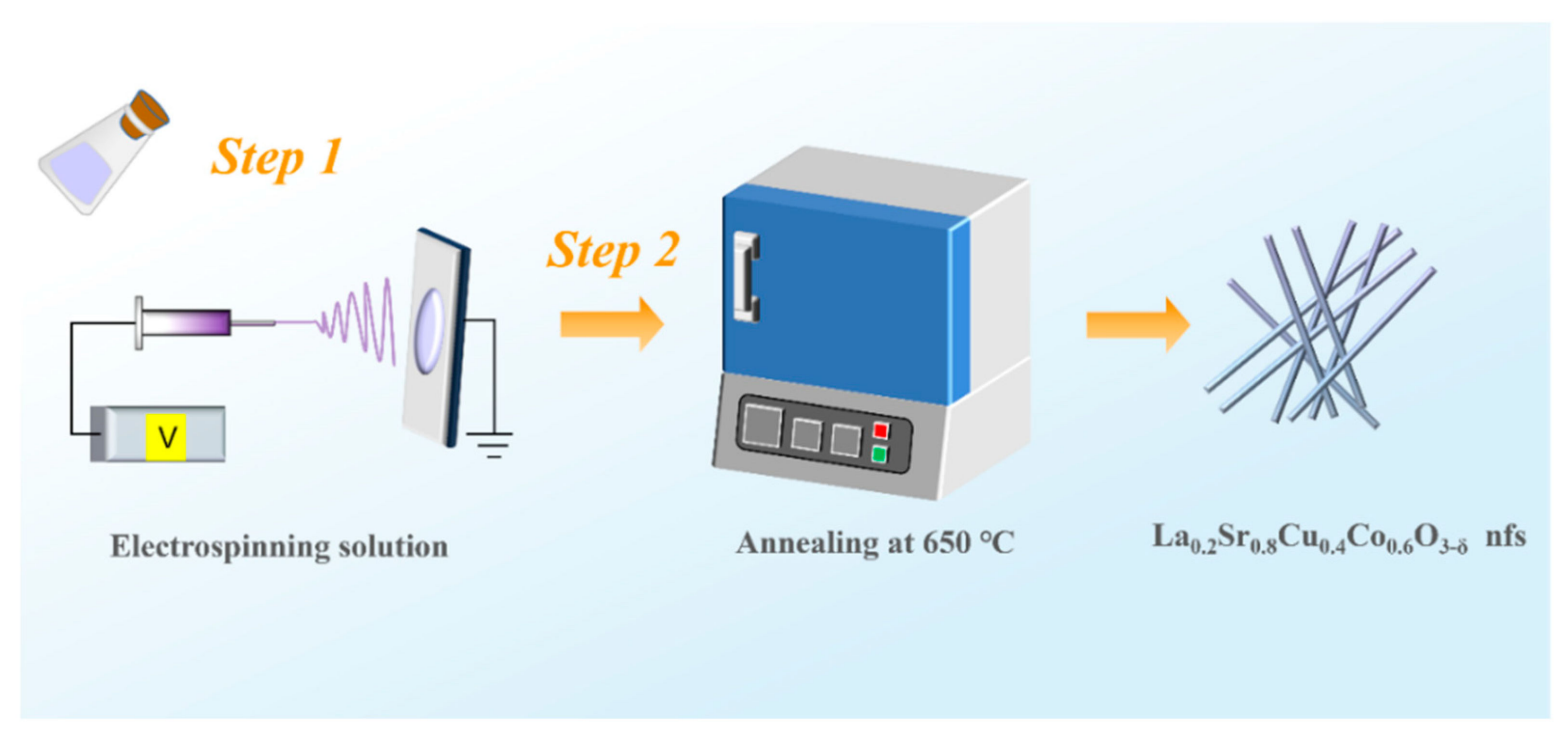
Schematic illustration of the synthesis processes of LSCC NFs.

The steps used to prepare the LSCC multi-particle nanochain structure are shown in Scheme 1. First, nanofibers were prepared by electrospinning. The electrospun nanofibers were then annealed in air (500–800 °C) to obtain LSCC NFs. The formation of nanofibers was promoted in the strong oxidizing environment during calcination. The nanofiber structure consisted of well-dispersed nanoparticles forming a smooth surface.

**Figure 1:**
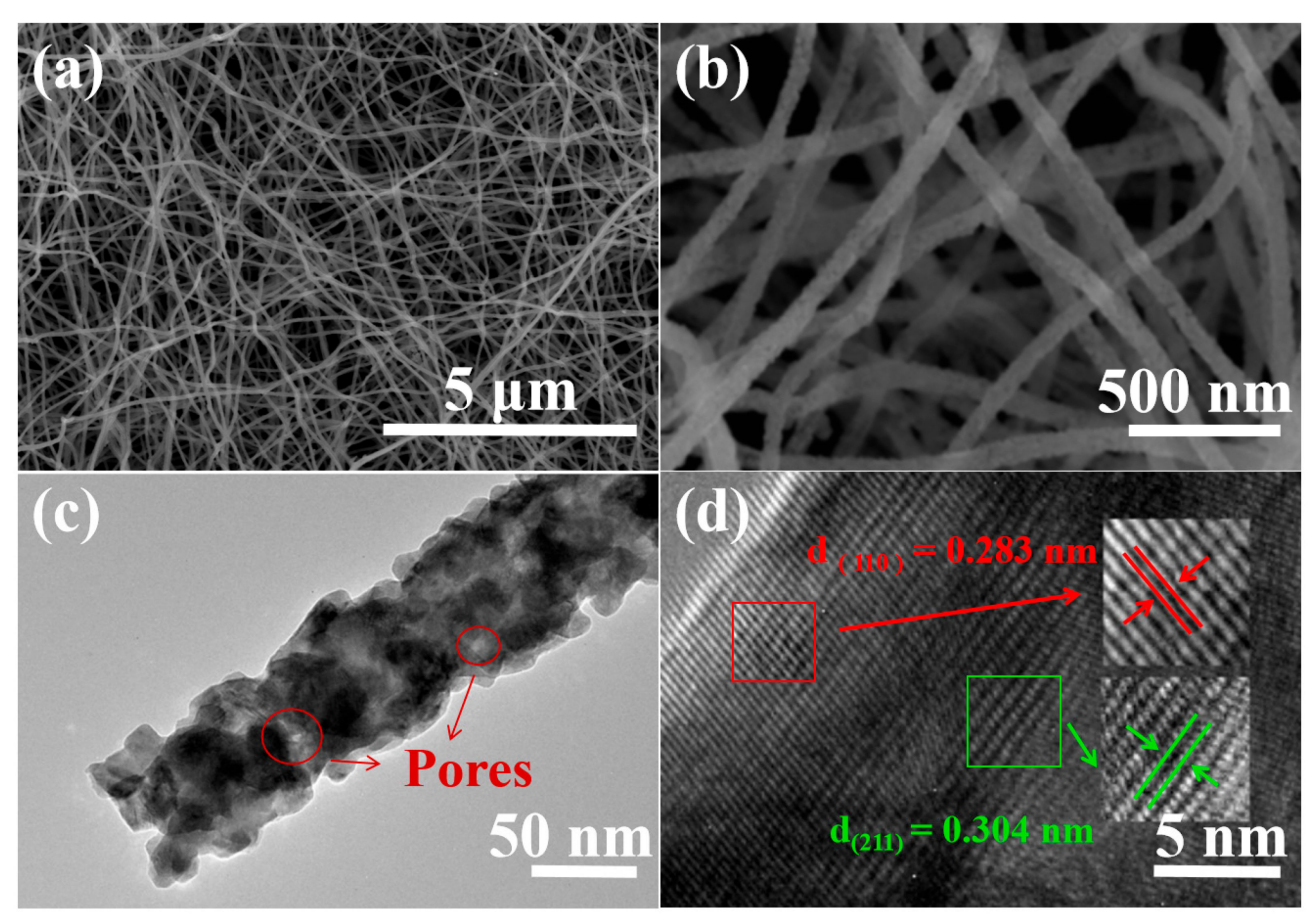
Scanning electron microscope (SEM) images of (**a**,**b**) LSCC-650; (**c**,**d**) TEM image of LSCC-650.

**Figure 2:**
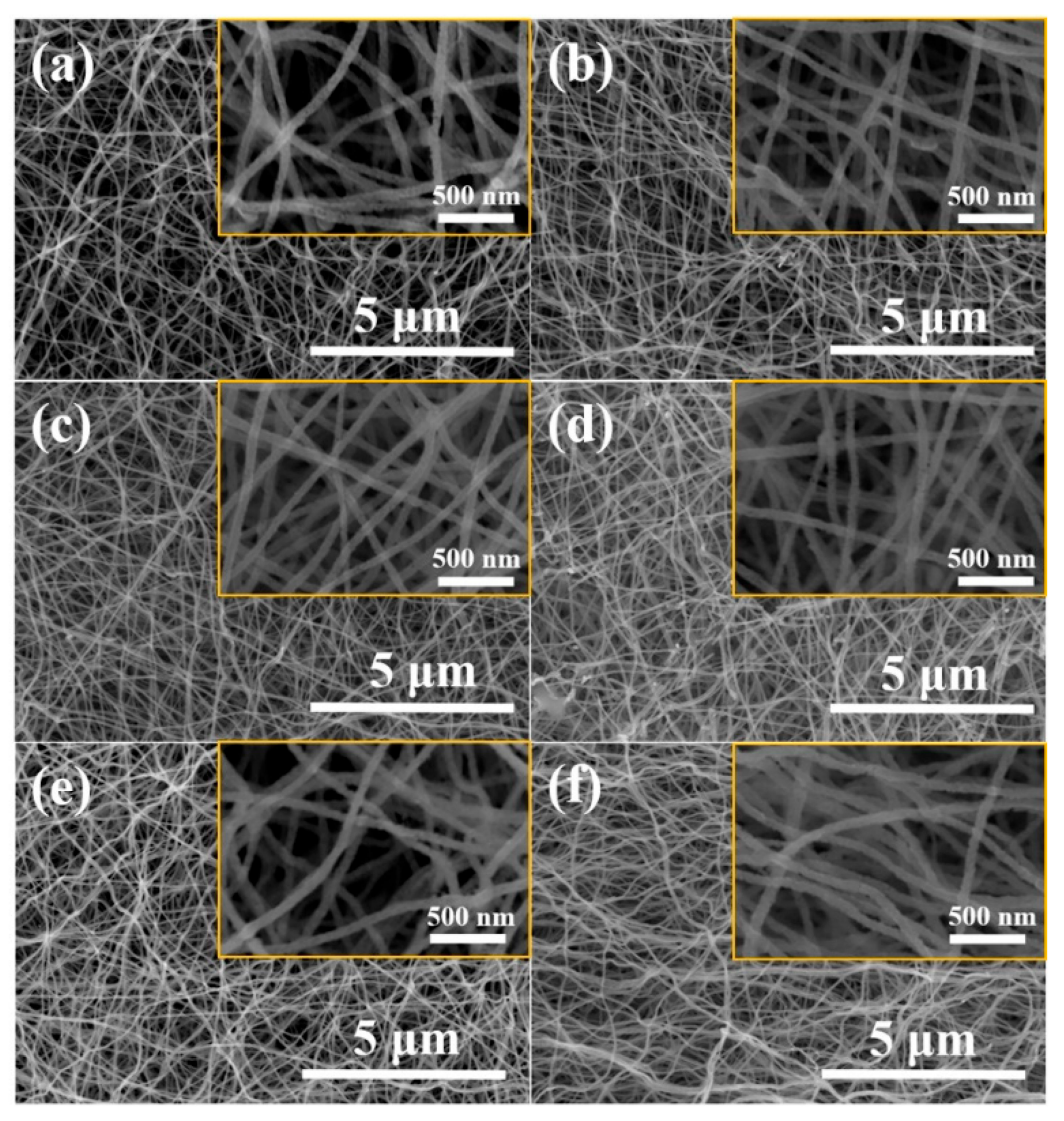
SEM images of (**a**) LSCC-500, (**b**) LSCC-550, (**c**) LSCC-600, (**d**) LSCC-700, (**e**) LSCC-750, and (**f**) LSCC-800.

The diameter and shape of the LSCC nanofibers calcined at 500–800 °C were evaluated by SEM and TEM, as shown in Figure 1. Low- and high-magnification SEM images of LSCC-650 NFs (prepared by calcining at 650 °C) are shown in Figure 1a,b, respectively. A TEM image showing the microstructure of LSCC-650 NFs is displayed in Figure 1c. The LSCC-650 NF sample is composed of nanoparticles and shows many pores, which are marked by red circles. This high porosity provides a larger surface area for the reaction. The presence of pores on the nanofiber wall is due to the release of decomposition gases during calcination. The high-resolution TEM image shown in Figure 1d shows a lattice stripe spacing of approximately 0.283 and 0.304 nm, corresponding to the (110) and (211) lattice planes of LSCC-650 NF, respectively. After calcination at different temperatures, varying morphologies were obtained (Figure 2a–f). SEM images show that the obtained nanofibers generally exhibit diameters in the range of 160–220 nm, and the diameter slightly decreases with increasing calcination temperature from 500 to 800 °C. Moreover, the surface particle size increases with increasing calcination temperature. Calcination at temperatures higher or lower than 650 °C still results in the formation of some small pores, but these pores are not as obvious as those on the LSCC-650 NF surface. As a result, residual carbon forms and impedes particle growth, causing the LSCC NFs prepared at other calcination temperatures to have a more compact appearance. EDX analysis was used to confirm the presence of carbon residue in the samples (Supporting Information). As shown in Figures S2–S8, the carbon content of the samples decreases with increasing calcination temperature. After calcination at 450 °C, LSCC-500 NFs had a carbon content of 81.02%. However, when the calcination temperature was raised to 650 °C, the carbon content of LSCC-650 NFs decreased to 19.70%. This suggests that reducing the carbon content is conducive to enhancing the catalytic performance of the perovskite component of the samples, leading to improved OER activity.

**Figure 3:**
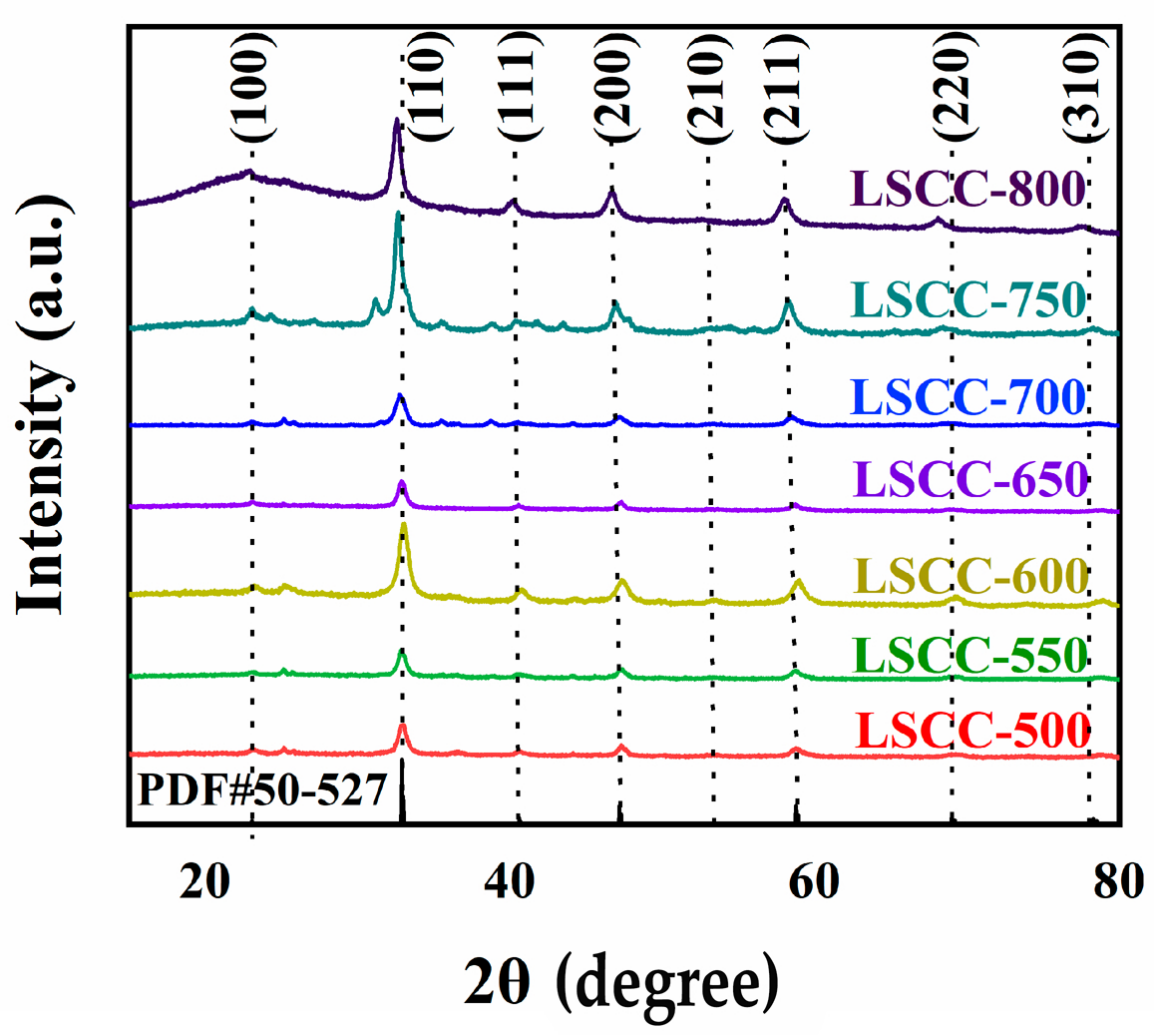
The XRD patterns of the LSCC nanofibers produced in different proportions.

XRD patterns of LSCC-500 NFs, LSCC-550 NFs, LSCC-600 NFs, LSCC-650 NFs, LSCC-700 NFs, LSCC-750 NFs, and LSCC-500 NFs are shown in Figure 3. All diffraction peaks are consistent with those of La_0.2_Sr_0.8_Cu_0.4_Co_0.6_O_3−δ_ perovskite-type oxides (JCPDS No. 50-527). These diffraction patterns indicate that the LSCC crystal structure is not as well-formed at low calcination temperatures. Moreover, LSCC-750 NFs (calcined at 750 °C) also show the presence of impurities that are absent from the pure LSCC phase. These results indicate that the calcination temperature for preparing LSCC nanofibers should be lower than 750 °C. Increasing the calcination temperature to 750 °C leads to a corresponding increase in diffraction peak intensity. These XRD patterns show that the perovskite grain size increases with increasing calcination temperature. According to some research reports, smaller crystals contain more oxygen vacancies, leading to better OER performance [60,61]. As demonstrated by its XRD pattern, LSCC-650 NFs consist of a pure LSCC phase with a small grain size. Thus, LSCC-650 NFs are expected to have the highest oxygen vacancy concentration and the best OER performance.

**Figure 4:**
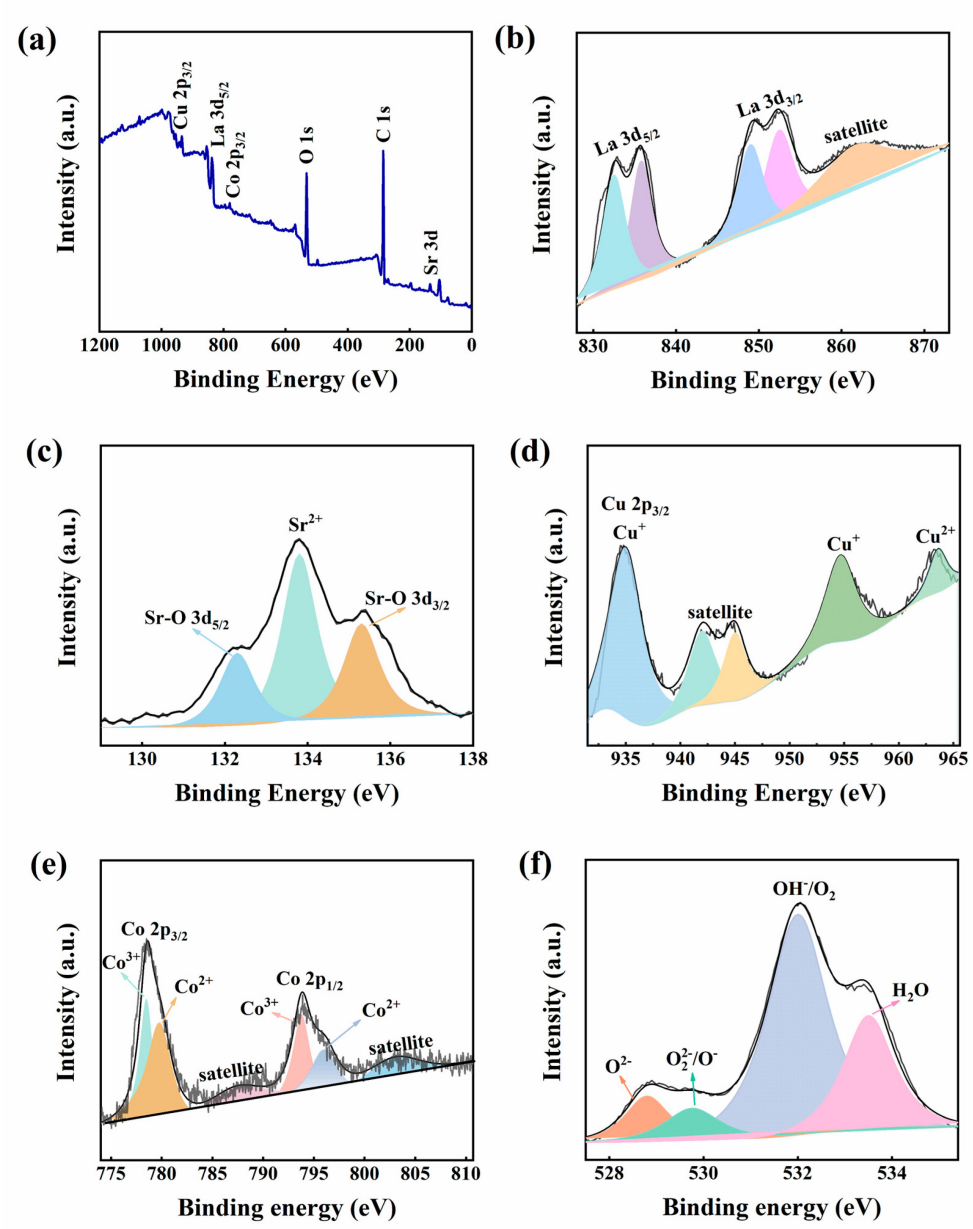
High resolution XPS spectrum of (**a**) LSCC, (**b**) La 3d_3/2_, (**c**) Sr_3/2_, (**d**) Cu 2p_3/2_, (**e**) Co 2p_3/2_, and (**f**) O 1s in La_0.2_Sr_0.8_Cu_0.4_Co_0.6_O_3−δ_ oxide.

The surface chemical properties of LSCC-650 NFs were evaluated by XPS. The survey spectrum of LSCC-650 NFs is shown in Figure 4a, and the high-resolution La 3d, Sr 3d, Co 2p, Cu 2p, and O 1s spectra are shown in Figure 4b–f. As displayed in Figure 4b, the La 3d spectrum exhibits four prominent bands. The peaks at 832.5 and 835.8 eV correspond to La 3d_5/2_, while those at 849.0 and 852.5 eV correspond to La 3d_3/2_. These bands reveal that the La in LSCC-650 NFs is mostly in a metallic state [62,63,64]. As shown in Figure 4c, the Sr 3d spectrum shows two peaks at 132.2 eV and 135.5 eV corresponding to the Sr-O bond (Sr-O 3d_5/2_ and Sr-O 3d_3/2_, respectively). Moreover, a peak at 133.8 eV corresponds to Sr^2+^ [65,66]. The Cu 2p spectrum shown in Figure 4d shows three main peaks. The peaks at 935.1 and 954.5 eV correspond to Cu^+^, while the peak at 963.6 eV is assigned to Cu^2+^ [67]. The deconvoluted Co 2p spectrum (Figure 4e) displays four prominent bands and two satellite peaks. The main peaks at 780.0 and 796.8 eV are ascribed to Co^2+^, while the peaks at 778.3 and 794.2 eV are ascribed to Co^3+^ [68,69,70]. The O 1s spectrum shown in Figure 4f contains four characteristic peaks at 528.2, 529.4, 532.1, and 533.6 eV corresponding to lattice oxygen (O^2−^), adsorbed oxidative oxygen species (O^−^/O^2−^_2_), adsorbed molecular oxygen and hydroxyl groups (O_2_/OH^−^), and adsorbed water molecules (H_2_O). Among them, O^−^/O^2−^_2_ are active sites for OER in alkaline solutions, and the presence of these sites has a positive impact on oxygen transport [71,72,73].

**Figure 5:**
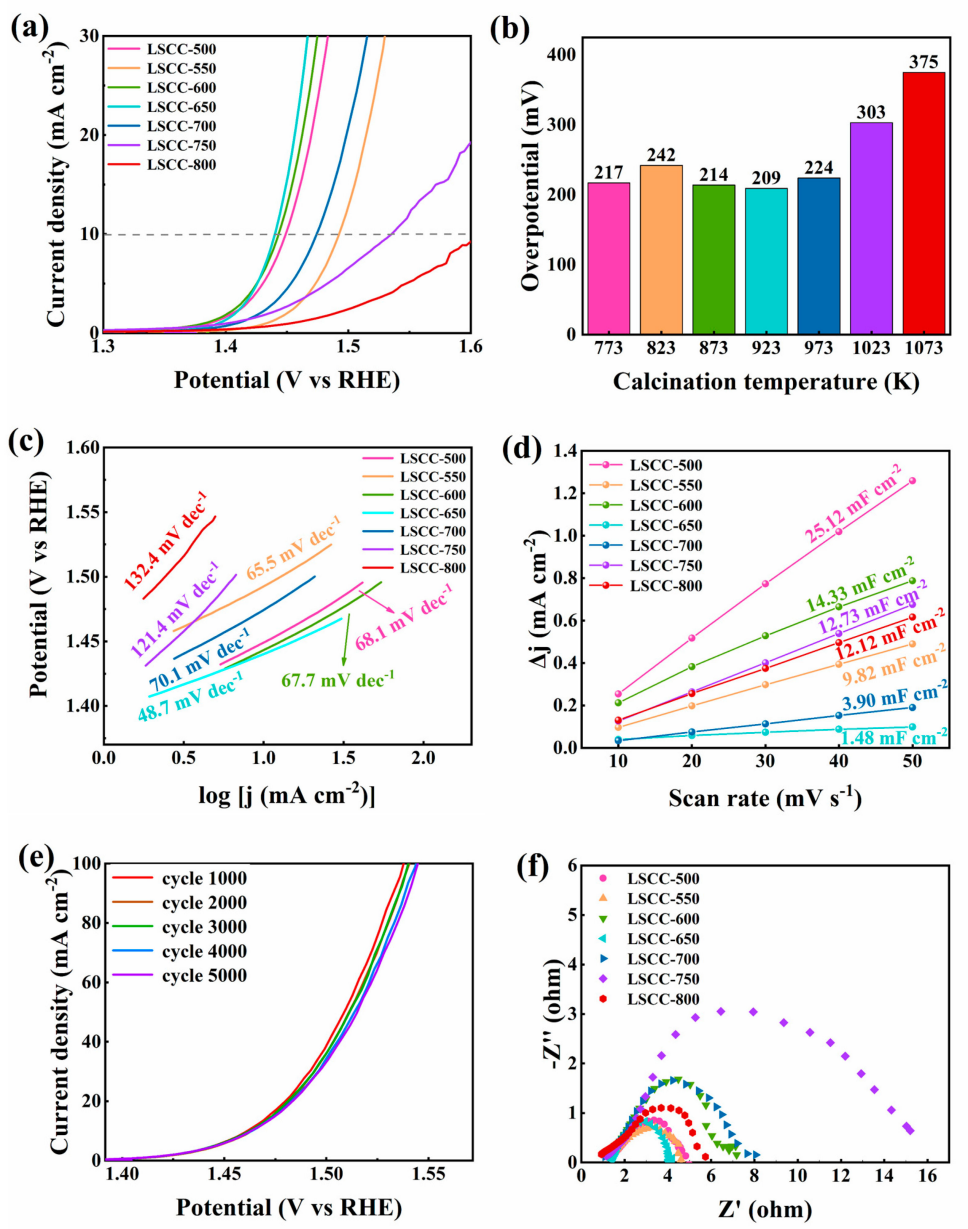
Electrocatalytic OER performance of the LSCC nanofibers produced in different temperatures. (**a**) LSV curves of the prepared electrodes in 6.0 M KOH. (**b**) For comparison, the corresponding overpotential of the synthesized catalysts realized a current density of 10 mA cm^−2^. (**c**) Comparison of the Tafel plots of synthesized catalysts. (**d**) The capacitive current plots as a function of the scan rate. (**e**) LSV curves of LSCC-650 NFs before and after 5000 cycles. (**f**) EIS pattern of the LSCC produced in different proportions researched at a fixed potential of 0.5 V (vs. SCE).

The electrochemical OER activity of the prepared catalysts was measured in 6.0 M KOH. The polarization curves of these nanofibers are shown in Figure 5a. At a specific current density of j = 10 mA cm^−2^, LSCC-650 NFs exhibit the lowest overpotential of 209 mV. This performance is superior to that of LSCC-500 NFs (217 mV), LSCC-550 NFs (260 mV), LSCC-600 NFs (214 mV), LSCC-700 NFs (242 mV), LSCC-750 NFs (303 mV), and LSCC-800 NFs (375 mV), as shown in Figure 5b. The reaction kinetic rates of these samples were also expressed by their Tafel slopes, as shown in Figure 5c. LSCC-650 NF show the lowest Tafel slope (48.7 mV dec^−1^) compared to LSCC-500 NFs (68.1 mV dec^−1^), LSCC-550 NFs (65.5 mV dec^−1^), LSCC-600 NFs (67.7 mV dec^−1^), LSCC-700 NFs (70.1 mV dec^−1^), LSCC-750 NFs (121.4 mV dec^−1^), and LSCC-800 NFs (132.4 mV dec^−1^). These results demonstrate that the OER performance of LSCC NFs declines as the calcination temperature is raised above 650 °C. This is because the size and specific surface area of the perovskite crystals increases with increasing calcination temperature, removing the advantageous properties of the nanoparticles. LSCC-650 NF show intact nanofibers and a well-retained structure after the reaction, which is one of the reasons for its high OER performance (Figure S9). Thus, due to its hierarchical structure, the OER activity of the prepared LSCC-650 NFs compares favorably with other reported perovskite OER electrocatalysts (Table S1).

The C_dl_ values of the prepared catalysts were calculated to evaluate their ECSAs. The CV curves of the LSCC NFs were obtained at different scanning rates, and the activity of the bilayer capacitor was calculated. The scanning potential was set to 0–0.1 V vs. RHE. As shown in Figure 5d, LSCC-650 NFs (1.48 mF cm^−2^) are superior compared to the other samples, proving that the LSCC-650 NF electrode has the most OER active sites. The long-term OER stability of LSCC-650 NFs was evaluated in a cycling test, as shown in Figure 5e. After every 1000 cycles, OER activity only slightly declined. After 5000 cycles, a decline of just 0.86% can be observed, confirming the excellent OER stability of this catalyst. This excellent stability demonstrates that, even after electrolysis, the LSCC-650 NF electrocatalyst can still maintain its nanofiber structure and electrocatalytic properties. Therefore, LSCC-650 NFs are suitable as a functional OER electrocatalyst.

## 4. Conclusions

In summary, LSCC NFs were prepared by a simple and easily scalable electrospinning technique followed by calcination. The obtained LSCC NFs showed good OER electrochemical performance, with LSCC-650 NFs exhibiting a low overpotential of just 209 mV at 100 mA cm^−2^. After 48 h of cycling (5000 cycles), only a slight change in overpotential was observed, demonstrating the good electrochemical stability of LSCC-650 NFs. Overall, the prepared catalysts exhibit good durability and performance due to their nanofiber structure. Moreover, these catalysts were synthesized using low-cost non-noble metals. Therefore, they show excellent promise for use as OER electrodes in water-splitting applications. In future work, the LSCC content of the catalysts will be enhanced to further improve their electrochemical performance.

## Data Availability

All the relevant data are included in this published article.

## Supplementary Materials

The following supporting information can be downloaded at https://www.mdpi.com/article/10.3390/nano14010064/s1, Figure S1: OER reaction path. Blue line-acidic; red line-alkaline; Black Line-OOH_ad_ path; Green Line-2O_ads_ directly combine to produce O_2_; Figure S2: EDX of the LSCC-500 nanofibers; Figure S3: EDX of the LSCC-550 nanofibers; Figure S4: EDX of the LSCC-600 nanofibers; Figure S5. EDX of the LSCC-650 nanofibers; Figure S6: EDX of the LSCC-700 nanofibers; Figure S7: EDX of the LSCC-750 nanofibers; Figure S8: EDX of the LSCC-800 nanofibers; Figure S9: SEM figure after 5000 cycles images of the LSCC-650 nanofibers; Table S1: Summary of OER overpotentials at 10 mA cm^−2^ for perovskite oxides-based electrocatalysts obtained through composition engineering. References [38,39,40,73,74,75,76,77,78,79,80,81,82,83,84,85,86,87,88,89,90,91,92,93,94,95] are cited in the Supplementary Materials.

## References

[1] Li M., Bi X., Wang R., Li Y., Jiang G., Li L., Zhong C., Chen Z., Lu J. (2020). Relating catalysis between fuel cell and metal-air batteries. Matter.

[2] Wen X., Zhang Q., Guan J. (2020). Applications of metal–organic framework-derived materials in fuel cells and metal-air batteries. Coord. Chem. Rev..

[3] Zhang S., Chen M., Zhao X., Cai J., Yan W., Yen J.C., Chen S., Yu Y., Zhang J. (2021). Advanced noncarbon materials as catalyst supports and non-noble electrocatalysts for fuel cells and metal-air batteries. Electrochem. Energy Rev..

[4] Lu L., Zheng Y., Yang R., Kakimov A., Li X. (2021). Recent advances of layered double hydroxides–based bifunctional electrocatalysts for ORR and OER. Mater. Today Chem..

[5] Liu M., Zhao Z., Duan X., Huang Y. (2019). Nanoscale structure design for high-performance Pt-based ORR catalysts. Adv. Mater..

[6] Wang X., Zhong H., Xi S., Lee W.S.V., Xue J. (2022). Understanding of oxygen redox in the oxygen evolution reaction. Adv. Mater..

[7] Xie X., Du L., Yan L., Park S., Qiu Y., Sokolowski J., Wang W., Shao Y. (2022). Oxygen evolution reaction in alkaline environment: Material challenges and solutions. Adv. Funct. Mater..

[8] Gao F., He J., Wang H., Lin J., Chen R., Yi K., Huang F., Lin Z., Wang M. (2022). Te-mediated electro-driven oxygen evolution reaction. Nano Res. Energy.

[9] Haase F.T., Bergmann A., Jones T.E., Timoshenko J., Herzog A., Jeon H.S., Rettenmaier C., Cuenya B.R. (2022). Size effects and active state formation of cobalt oxide nanoparticles during the oxygen evolution reaction. Nat. Energy.

[10] Guo T., Li L., Wang Z. (2022). Recent development and future perspectives of amorphous transition metal-based electrocatalysts for oxygen evolution reaction. Adv. Energy Mater..

[11] Xu H., Yuan J., He G., Chen H. (2023). Current and future trends for spinel-type electrocatalysts in electrocatalytic oxygen evolution reaction. Coord. Chem. Rev..

[12] Wang C., Jin L., Shang H., Xu H., Shiraishi Y., Du Y. (2021). Advances in engineering RuO_2_ electrocatalysts towards oxygen evolution reaction. Chin. Chem. Lett..

[13] Schweinar K., Gault B., Mouton I., Kasian O. (2020). Lattice oxygen exchange in rutile IrO_2_ during the oxygen evolution reaction. J. Phys. Chem. Lett..

[14] Hu Z., Hao L., Quan F., Guo R. (2022). Recent developments of Co_3_O_4_-based materials as catalysts for the oxygen evolution reaction. Catal. Sci. Technol..

[15] Peng X., Feng S., Lai S., Liu Z., Gao J., Javanbakht M., Gao B. (2022). Structural engineering of rare-earth-based perovskite electrocatalysts for advanced oxygen evolution reaction. Int. J. Hydrogen Energy.

[16] Zhao J.-W., Zhang H., Li C.-F., Zhou X., Wu J.-Q., Zeng F., Zhang J., Li G.-R. (2022). Key roles of surface Fe sites and Sr vacancies in the perovskite for an efficient oxygen evolution reaction via lattice oxygen oxidation. Energy Environ. Sci..

[17] Liu Y., Ye C., Zhao S.-N., Wu Y., Liu C., Huang J., Xue L., Sun J., Zhang W., Wang X. (2022). A dual-site doping strategy for developing efficient perovskite oxide electrocatalysts towards oxygen evolution reaction. Nano Energy.

[18] Tomar A.K., Pan U.N., Kim N.H., Lee J.H. (2022). Enabling Lattice Oxygen Participation in a Triple Perovskite Oxide Electrocatalyst for the Oxygen Evolution Reaction. ACS Energy Lett..

[19] Liu C., Ji D., Shi H., Wu Z., Huang H., Kang Z., Chen Z. (2022). An A-site management and oxygen-deficient regulation strategy with a perovskite oxide electrocatalyst for the oxygen evolution reaction. J. Mater. Chem. A.

[20] Xie J., Gao Y., Chen G., Wang Y., Yu J., Ciucci F., Chen D., Shao Z. (2022). Simultaneously improved surface and bulk participation of evolved perovskite oxide for boosting oxygen evolution reaction activity using a dynamic cation exchange strategy. Small.

[21] Wang Q., Wang H., Qi S., Su Z., Chen K., Yu X., Xie A., Luo S. (2022). Coral-Like LaNi_x_Fe_1− x_O_3_ perovskite catalyst for high-performance oxygen evolution reaction. J. Electrochem. Soc..

[22] Jo H., Yang Y., Seong A., Jeong D., Kim J., Joo S.H., Kim Y.J., Zhang L., Liu Z., Wang J.-Q. (2022). Promotion of the oxygen evolution reaction via the reconstructed active phase of perovskite oxide. J. Mater. Chem. A.

[23] He X., Qiao T., Li B., Zhang Z., Wang S., Wang X., Liu H. (2023). Tuning Electronic Structure of CuCo_2_O_4_ Spinel via Mn-Doping for Enhancing Oxygen Evolution Reaction. ChemElectroChem.

[24] Olowoyo J.O., Kriek R.J. (2022). Recent Progress on Bimetallic-Based Spinels as Electrocatalysts for the Oxygen Evolution Reaction. Small.

[25] Xiang W., Yang N., Li X., Linnemann J., Hagemann U., Ruediger O., Heidelmann M., Falk T., Aramini M., DeBeer S. (2022). 3D atomic-scale imaging of mixed Co-Fe spinel oxide nanoparticles during oxygen evolution reaction. Nat. Commun..

[26] Avcı O.N., Sementa L., Fortunelli A. (2022). Mechanisms of the Oxygen Evolution reaction on NiFe_2_O_4_ and CoFe_2_O_4_ inverse-spinel oxides. ACS Catal..

[27] Wang Z., Huang J., Wang L., Liu Y., Liu W., Zhao S., Liu Z.Q. (2022). Cation-tuning induced d-band center modulation on Co-based spinel oxide for oxygen reduction/evolution reaction. Angew. Chem..

[28] Talluri B., Yoo K., Kim J. (2022). High entropy spinel metal oxide (CoCrFeMnNi)_3_O_4_ nanoparticles as novel efficient electrocatalyst for methanol oxidation and oxygen evolution reactions. J. Environ. Chem. Eng..

[29] Cui M., Yang C., Li B., Dong Q., Wu M., Hwang S., Xie H., Wang X., Wang G., Hu L. (2021). High-entropy metal sulfide nanoparticles promise high-performance oxygen evolution reaction. Adv. Energy Mater..

[30] He R., Huang X., Feng L. (2022). Recent progress in transition-metal sulfide catalyst regulation for improved oxygen evolution reaction. Energy Fuels.

[31] Peng L., Shah S.S.A., Wei Z. (2018). Recent developments in metal phosphide and sulfide electrocatalysts for oxygen evolution reaction. Chin. J. Catal..

[32] Sivanantham A., Ganesan P., Vinu A., Shanmugam S. (2019). Surface activation and reconstruction of non-oxide-based catalysts through in situ electrochemical tuning for oxygen evolution reactions in alkaline media. Acs Catal..

[33] Li W., Xiong D., Gao X., Liu L. (2019). The oxygen evolution reaction enabled by transition metal phosphide and chalcogenide pre-catalysts with dynamic changes. Chem. Commun..

[34] Dai W., Bai X., Zhu Y.-a., Zhang Y., Lu T., Pan Y., Wang J. (2021). Surface reconstruction induced in situ phosphorus doping in nickel oxides for an enhanced oxygen evolution reaction. J. Mater. Chem. A.

[35] Gao L., Cui X., Sewell C.D., Li J., Lin Z. (2021). Recent advances in activating surface reconstruction for the high-efficiency oxygen evolution reaction. Chem. Soc. Rev..

[36] Huang W., Chen C., Ling Z., Li J., Qu L., Zhu J., Yang W., Wang M., Owusu K.A., Qin L. (2021). Ni/Fe based bimetallic coordination complexes with rich active sites for efficient oxygen evolution reaction. Chem. Eng. J..

[37] Li X., Wang C., Xue H., Pang H., Xu Q. (2020). Electrocatalysts optimized with nitrogen coordination for high-performance oxygen evolution reaction. Coord. Chem. Rev..

[38] Zhang Z., Chen Y., Li H., Hua H. (2011). Simulation and experimental study on vibration and sound radiation control with piezoelectric actuators. Shock Vib..

[39] Liu H., Xie R., Wang Q., Han J., Han Y., Wang J., Fang H., Qi J., Ding M., Ji W. (2023). Enhanced OER Performance and Dynamic Transition of Surface Reconstruction in LaNiO_3_ Thin Films with Nanoparticles Decoration. Adv. Sci..

[40] Li L., Jiang B., Tang D., Zhang Q., Zheng Z. (2018). Hydrogen generation by acetic acid steam reforming over Ni-based catalysts derived from La_1−x_Ce_x_NiO_3_ perovskite. Int. J. Hydrogen Energy.

[41] Torregrosa-Rivero V., Sanchez-Adsuar M.-S., Illan-Gomez M.-J. (2022). Analyzing the role of copper in the soot oxidation performance of BaMnO_3_-perovskite-based catalyst obtained by modified sol-gel synthesis. Fuel.

[42] Navas D., Fuentes S., Castro-Alvarez A., Chavez-Angel E. (2021). Review on sol-gel synthesis of perovskite and oxide nanomaterials. Gels.

[43] Yu L., Xu N., Zhu T., Xu Z., Sun M., Geng D. (2020). La_0_._4_Sr_0_._6_Co_0_._7_Fe_0_._2_Nb_0_._1_O_3−δ_ perovskite prepared by the sol-gel method with superior performance as a bifunctional oxygen electrocatalyst. Int. J. Hydrogen Energy.

[44] Karami M., Ghanbari M., Amiri O., Salavati-Niasari M. (2020). Enhanced antibacterial activity and photocatalytic degradation of organic dyes under visible light using cesium lead iodide perovskite nanostructures prepared by hydrothermal method. Sep. Purif. Technol..

[45] Walton R.I. (2020). Perovskite oxides prepared by hydrothermal and solvothermal synthesis: A review of crystallisation, chemistry, and compositions. Chem.–A Eur. J..

[46] Wei L., Zhang Y., Zhang C., Yao C., Ni C., Li X. (2023). In Situ Growth of Perovskite on 2D Hydrothermal Carbonation Carbon for Photocatalytic Reduction of Nitrate to Ammonia. ACS Appl. Nano Mater..

[47] Vu T.H.Q., Bondzior B., Stefańska D., Miniajluk N., Dereń P.J. (2020). Synthesis, structure, morphology, and luminescent properties of Ba_2_Mg_W_O_6_: Eu^3+^ double perovskite obtained by a novel Co-precipitation method. Materials.

[48] Nguyen T.A., Pham V., Pham T.L., Nguyen L.T.T., Mittova I.Y., Mittova V., Vo L.N., Nguyen B.T.T., Bui V.X., Viryutina E. (2020). Simple synthesis of NdFeO_3_ nanoparticles by the co-precipitation method based on a study of thermal behaviors of Fe (III) and Nd (III) hydroxides. Crystals.

[49] Wang Y., Jiang Y., Zhao Y., Ge X., Lu Q., Zhang T., Xie D., Li M., Bu Y. (2023). Design strategies of perovskite nanofibers electrocatalysts for water splitting: A mini review. Chem. Eng. J..

[50] Wu X., Miao H., Hu R., Chen B., Yin M., Zhang H., Xia L., Zhang C., Yuan J. (2021). A-site deficient perovskite nanofibers boost oxygen evolution reaction for zinc-air batteries. Appl. Surf. Sci..

[51] Bkkar M., Olekhnovich R., Kremleva A., Sitnikova V., Kovach Y., Zverkov N., Uspenskaya M. (2023). Influence of electrospinning setup parameters on properties of polymer-perovskite nanofibers. Polymers.

[52] Zhao B., Gao X., Pan K., Deng J. (2021). Chiral helical polymer/perovskite hybrid nanofibers with intense circularly polarized luminescence. ACS Nano.

[53] Li G., Hou S., Gui L., Feng F., Zhang D., He B., Zhao L. (2019). Carbon quantum dots decorated Ba_0_._5_Sr_0_._5_Co_0_._8_Fe_0_._2_O_3−δ_ perovskite nanofibers for boosting oxygen evolution reaction. Appl. Catal. B Environ..

[54] Chen L., Chuang Y., Yang W.-D., Tsai K.-C., Chen C.-W., Dong C.-D. (2021). All-inorganic perovskite CsPbX_3_ electrospun nanofibers with color-tunable photoluminescence and high-performance optoelectronic applications. J. Alloys Compd..

[55] Fu L., Zhou J., Deng Q., Yang J., Li Q., Zhu Z., Wu K. (2023). Interfacial electron transfer in heterojunction nanofibers for highly efficient oxygen evolution reaction. Nanoscale.

[56] Wei P., Sun X., Liang Q., Li X., He Z., Hu X., Huang Y. (2020). Enhanced oxygen evolution reaction activity by encapsulating NiFe alloy nanoparticles in nitrogen-doped carbon nanofibers. ACS Appl. Mater. Interfaces.

[57] Lin H., Xie J., Zhang Z., Wang S., Chen D. (2021). Perovskite nanoparticles@ N-doped carbon nanofibers as robust and efficient oxygen electrocatalysts for Zn-air batteries. J. Colloid Interface Sci..

[58] El-Shazly A.N., Rezk M.Y., Gameel K.M., Allam N.K. (2019). Electrospun lead-free all-inorganic double perovskite nanofibers for photovoltaic and optoelectronic applications. ACS Appl. Nano Mater..

[59] Fu Y., Guo L., Ren Z., Li X., Zhang Q., Wu J., Li Y., Liu W., Li P., Ma J. (2023). Enhanced property of flexible UV photodetectors based on electrospinning ZnO-SnO_2_ heterojunction nanofibers by the formation of Zn_2_SnO_4_. Ceram. Int..

[60] Bathula C., Jana A., Soni R., Naushad M., Kim H.-S. (2023). Enhanced stability of methylammonium lead bromide perovskite interlaced on cellulose nanofiber. Ceram. Int..

[61] Béjar J., Ãlvarez-Contreras L., Ledesma-GarcÃa J., Arjona N., Arriaga L. (2019). Electrocatalytic evaluation of Co_3_O_4_ and NiCo_2_O_4_ rosettes-like hierarchical spinel as bifunctional materials for oxygen evolution (OER) and reduction (ORR) reactions in alkaline media. J. Electroanal. Chem..

[62] Sun B., Liu Y., Hu Y., Zhou H., Zhang Z., Wang J., Gu S., Shen Y. (2023). Hygroscopic La_2_O_3_ hybridized LaCoO_3_-based composite catalyst for efficient ozone decomposition under humidity conditions. Appl. Surf. Sci..

[63] Lv C., Chen H., Hu M., Ai T., Fu H. (2021). Nano-oxides washcoat for enhanced catalytic oxidation activity toward the perovskite-based monolithic catalyst. Environ. Sci. Pollut. Res..

[64] Natile M.M., Ugel E., Maccato C., Glisenti A. (2007). LaCoO_3_: Effect of synthesis conditions on properties and reactivity. Appl. Catal. B Environ..

[65] Mandal R., Mahton Y., Sowjanya C., Sanket K., Behera S.K., Pratihar S.K. (2023). Electrocatalytic behaviour of Cu-substituted La_0_._5_Sr_0_._5_Co_0_._8_Fe_0_._2−x_Cu_x_O_3−δ_ (x = 0–0.2) perovskite oxides. J. Solid State Chem..

[66] Zhang L., Zhu H., Hao J., Wang C., Wen Y., Li H., Lu S., Duan F., Du M. (2019). Integrating the cationic engineering and hollow structure engineering into perovskites oxides for efficient and stable electrocatalytic oxygen evolution. Electrochim. Acta.

[67] Yang J., Liu W., Xu M., Liu X., Qi H., Zhang L., Yang X., Niu S., Zhou D., Liu Y. (2021). Dynamic behavior of single-atom catalysts in electrocatalysis: Identification of Cu-N_3_ as an active site for the oxygen reduction reaction. J. Am. Chem. Soc..

[68] Li F., Tang J., Ke Q., Guo Y., Ha M.N., Wan C., Lei Z., Gu J., Ling Q., Nguyen V.N. (2021). Investigation into Enhanced Catalytic Performance for Epoxidation of Styrene over LaSrCo_x_Fe_2−x_O_6_ Double Perovskites: The Role of Singlet Oxygen Species Promoted by the Photothermal Effect. ACS Catal..

[69] Wen L., Zhang X., Liu J., Li X., Xing C., Lyu X., Cai W., Wang W., Li Y. (2019). Cr dopant induced breaking of scaling relations in Co Fe layered double hydroxides for improvement of oxygen evolution reaction. Small.

[70] Du Y., Ma W., Li H. (2020). In situ growth of CoP_3_/carbon polyhedron/CoO/NF nanoarrays as binderâ€ free anode for lithiumâ€ ion batteries with enhanced specific capacity. Small.

[71] Wang J., Yoshida A., Wang P., Yu T., Wang Z., Hao X., Abudula A., Guan G. (2020). Catalytic oxidation of volatile organic compound over cerium modified cobalt-based mixed oxide catalysts synthesized by electrodeposition method. Appl. Catal. B Environ..

[72] Zhao Y.-N., Liu C., Xu S., Min S., Wang W., Mitsuzaki N., Chen Z. (2023). A/B-Site Management Strategy to Boost Electrocatalytic Overall Water Splitting on Perovskite Oxides in an Alkaline Medium. Inorg. Chem..

[73] Zhu K., Shi F., Zhu X., Yang W. (2020). The roles of oxygen vacancies in electrocatalytic oxygen evolution reaction. Nano Energy.

[74] Li P., Wan X., Su J., Liu W., Guo Y., Yin H., Wang D. (2022). A single-phase FeCoNiMnMo high-entropy alloy oxygen evolution anode working in alkaline solution for over 1000 h. ACS Catal..

[75] Yu J., Chen G., Sunarso J., Zhu Y., Ran R., Zhu Z., Shao Z. (2016). Cobalt oxide and cobalt-graphitic carbon core–shell based catalysts with remarkably high oxygen reduction reaction activity. Adv. Sci..

[76] Alegre C., Busacca C., Di Blasi A., Di Blasi O., Aricò S., Antonucci A.V., Baglio V. (2020). Toward more efficient and stable bifunctional electrocatalysts for oxygen electrodes using FeCo_2_O_4_/carbon nanofiber prepared by electrospinning. Mater. Today Energy.

[77] Wang H., Xu W., Richins S., Liaw K., Yan L., Zhou M., Luo H. (2019). Polymer-assisted approach to LaCo_1-x_Ni_x_O_3_ network nanostructures as bifunctional oxygen electrocatalysts. Electrochim. Acta.

[78] He D., He G., Jiang H., Chen Z., Huang M. (2017). Enhanced durability and activity of the perovskite electrocatalyst Pr_0_._5_Ba_0_._5_CoO_3−δ_ by Ca doping for the oxygen evolution reaction at room temperature. Chem. Commun..

[79] Yi K., Sun L., Li Q., Xia T., Huo L., Zhao H., Li J., Lü Z., Bassat J.M., Rougier A. (2016). Effect of Nd-deficiency on electrochemical properties of NdBaCo_2_O_6−δ_ cathode for intermediate-temperature solid oxide fuel cells. Int. J. Hydrogen Energy.

[80] Yu J., Wang Z., Wang J., Zhong W., Ju M., Cai R., Qiu C., Long X., Yang S. (2020). The role of ceria in a hybrid catalyst toward alkaline water oxidation. ChemSusChem.

[81] Zhu P., Hu M., Deng Y., Wang C. (2016). One-pot fabrication of a novel agar-polyacrylamide/graphene oxide nanocomposite double network hydrogel with high mechanical properties. Adv. Eng. Mater..

[82] Xiao L., Liang Y., Li Z., Wu S., Luo S., Sun H., Zhu S., Cui Z. (2022). Amorphous FeNiNbPC nanoprous structure for efficient and stable electrochemical oxygen evolution. J. Colloid Interface Sci..

[83] Wang C., Wang R., Peng Y., Chen J., Chen Z., Yin H., Li J. (2020). Nb-incorporated Fe(oxy) hydroxide derived from structural transformation for efficient oxygen evolution electrocatalysis. J. Mater. Chem. A.

[84] Chen Q., Liu G., Liu S., Su H., Wang Y., Li J., Luo C. (2018). Remodeling the tumor microenvironment with emerging nanotherapeutics. Trends Pharmacol. Sci..

[85] Wang C., Cheng Y., Ianni E., Lin B. (2017). A highly active and stable La_0_._5_Sr_0_._5_Ni_0_._4_Fe_0_._6_O_3-δ_ perovskite electrocatalyst for oxygen evolution reaction in alkaline media. Electrochim. Acta.

[86] Retuerto M., Calle-Vallejo F., Pascual L., Lumbeeck G., Fernandez-Diaz M.T., Croft M., Gopalakrishnan J., Pena M.A., Hadermann J., Greenblatt M. (2019). La_1_._5_Sr_0_._5_NiMn_0_._5_Ru_0_._5_O_6_ double perovskite with enhanced ORR/OER bifunctional catalytic activity. ACS Appl. Mater. Interfaces.

[87] Kumar N., Kumar M., Nagaiah T., Siruguri V., Rayaprol S., Yadav A., Jha S.N., Bhattacharyya D., Paul A.K. (2020). Investigation of new B-site-disordered perovskite oxide CaLaScRuO_6+δ_: An efficient oxygen bifunctional electrocatalyst in a highly alkaline medium. ACS Appl. Mater. Interfaces.

[88] Sun Y., Li R., Chen X., Wu J., Xie Y., Wang X., Zhang Y. (2021). A-site management prompts the dynamic reconstructed active phase of perovskite oxide OER catalysts. Adv. Energy Mater..

[89] Duan Y., Yu Y., Hu J., Zheng S., Zhang T., Ding H., Hu B., Fu Q., Yu L., Zheng X. (2019). Scaled-up synthesis of amorphous NiFeMo oxides and their rapid surface reconstruction for superior oxygen evolution catalysis. Angew. Chem. Int. Ed..

[90] Ning M., Zhang F., Wu L., Xing X., Wang D., Song S., Zhou Q., Yu L., Bao J., Chen S. (2022). Boosting efficient alkaline fresh water and seawater electrolysis via electrochemical reconstruction. Energy Environ. Sci..

[91] Karthik N., Atchudan R., Edison T., Choi S. (2023). Insights of pristine stainless steel mesh oxygen evolution reaction in diverse concentrations of potassium hydroxide. Mater. Lett..

[92] Peng Q., Zhuang X., Wei L., Shi L., Isimjan T., Hou R., Yang X. (2022). Niobium-Incorporated CoSe_2_ Nanothorns with Electronic Structural Alterations for Efficient Alkaline Oxygen Evolution Reaction at High Current Density. ChemSusChem.

[93] Mondal R., Ratnawat H., Mukherjee S., Gupta A., Singh P. (2022). Investigation of the role of Sr and development of superior Sr-doped hexagonal BaCoO_3−δ_ perovskite bifunctional OER/ORR catalysts in alkaline media. Energy Fuels.

[94] Zhu C., Nobuta A., Nakatsugawa I., Akiyama T. (2013). Solution combustion synthesis of LaMO_3_ (M = Fe, Co, Mn) perovskite nanoparticles and the measurement of their electrocatalytic properties for air cathode. Int. J. Hydrogen Energy.

[95] Lu Y., Chien Y., Liu C., You T., Hu C. (2017). Active site-engineered bifunctional electrocatalysts of ternary spinel oxides, M_0.1_Ni_0.9_Co_2_O_4_ (M: Mn, Fe, Cu, Zn) for the air electrode of rechargeable zinc–air batteries. J. Mater. Chem. A.

