# Supplementary material for: One-Dimensional La0.2Sr0.8Cu0.4Co0.6O3−δ Nanostructures for Efficient Oxygen Evolution Reaction
Source: Nanomaterials (Basel). 2023 Dec 26;14(1):64. doi: 10.3390/nano14010064 (PMC10781154; doi:10.3390/nano14010064)
Supplement: Supplementary file 1 [file nanomaterials-14-00064-s001.zip › nanomaterials-2764264-supplementary.pdf]

Article

One-Dimensional $La_{0.2}Sr_{0.8}Cu_{0.4}Co_{0.6}O_{3-\delta}$ Nanostructures for Efficient Oxygen Evolution Reaction

Dongshuang Wu [1], Yidan Chen [1], Yuelei Bai [2], Chunncheng Zhu [1,*] and Mingyi Zhang [1,*]

[1] Key Laboratory for Photonic and Electronic Bandgap Materials, Ministry of Education, School of Physics and Electronic Engineering, Harbin Normal University, Harbin 150025, China
[2] National Key Laboratory of Science and Technology on Advanced Composites in Special Environments, Harbin Institute of Technology, Harbin 150001, China
* Correspondence: zhuccshs@163.com (C.Z.); zhangmingyi@hrbnu.edu.cn (M.Z.)

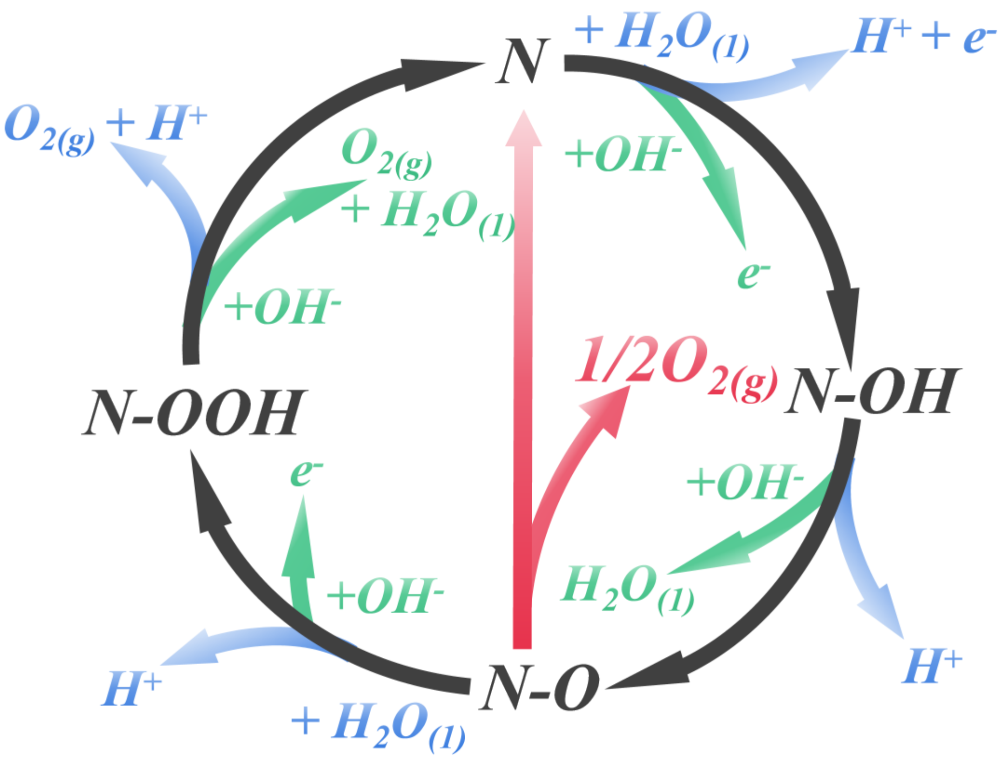

Figure S1. OER reaction path. Blue line-acidic; red line-alkaline; Black Line-OOH_{ad} path; Green Line-$2O_{ads}$ directly combine to produce O_2.

Catalysts	Electrolyte	Overpotential (mV) @10 mA cm^{-2}	Tafel slope [mV dec^{-1}]	References
$La_{0.2}Sr_{0.8}Cu_{0.4}Co_{0.6}O_{3-\delta}$	6 M KOH	209	48.7	this work
$La_{0.6}Sr_{0.4}CoO_3$	1 M KOH	426	—	[1]
FeCoNiMnMo	6 M KOH	279	56.1	[2]
$LaNiO_3$	1 M KOH	607	62	[3]
$La_{1-x}Ce_xNiO_3$	1 M KOH	370	69	[4]
$La_{0.2}Sr_{0.8}FeO_{3-\delta}$	0.1 M KOH	370	60	[5]
$FeCo_2O_4$	6 M KOH	130	—	[6]
$LaCo_{0.9}Ni_{0.1}O_3$	0.1 M KOH	650	73	[7]
$Pr_{0.5}Ba_{0.5}CoO_{3-\delta}$	0.1 M KOH	440	82	[8]
$PrBa_{0.25}Sr_{0.75}Co_2O_{5.95}$	0.1 M KOH	290	75.8	[9]
CeO_{2-x}	6 M KOH	777	76	[10]
$La_{0.6}Sr_{0.4}Co_{0.8}Fe_{0.2}O_3$	1 M KOH	353	63	[11]
FeNiNbPC	6 M KOH	248	40	[12]
$Fe_{90}Nb_{10}$	1 M KOH	380	40	[13]
$La_{0.8}Sr_{0.2}Co_{0.8}Fe_{0.2}O_{3-\delta}$	1 M KOH	248	51	[14]
$LaSr_3Co_{1.5}Fe_{1.5}O_{10-\delta}$	0.1 M KOH	388	84	[15]
$La_{0.5}Sr_{0.5}Ni_{0.4}Fe_{0.6}O_{3-\delta}$	1 M KOH	330	76	[16]
$CaLaScRuO_{6+\delta}$	1 M KOH	470	84	[17]
$La_{1.5}Sr_{0.5}NiMn_{0.5}Ru_{0.5}O_6$	0.1 M KOH	430	—	[18]
NiFeMo oxide	0.1 M KOH	280	49	[19]
$Fe_{0.01}$-Ni&$Ni_{0.2}Mo_{0.8}N$	6 M KOH	539	51	[20]
Stainless steel (Fe, Ni and Cr)	6 M KOH	223	44	[21]
Nb-$CoSe_2$	1 M KOH	297	54.1	[22]
Co_3O_4	6 M KOH	230	—	[23]
$BaCoO_{3-\delta}$	6 M KOH	395	60	[24]

$LaMnO_3$	6 M KOH	348	—	[25]
$LaCoO_3$	6 M KOH	362	—	[25]
$LaFeO_3$	6 M KOH	425	—	[25]
MnO_2-$LaCoO_3$	6 M KOH	442	—	[26]

Table S1. Summary of OER overpotentials at 10 mA cm^{-2} for perovskite oxides-based electrocatalysts obtained through composition engineering.

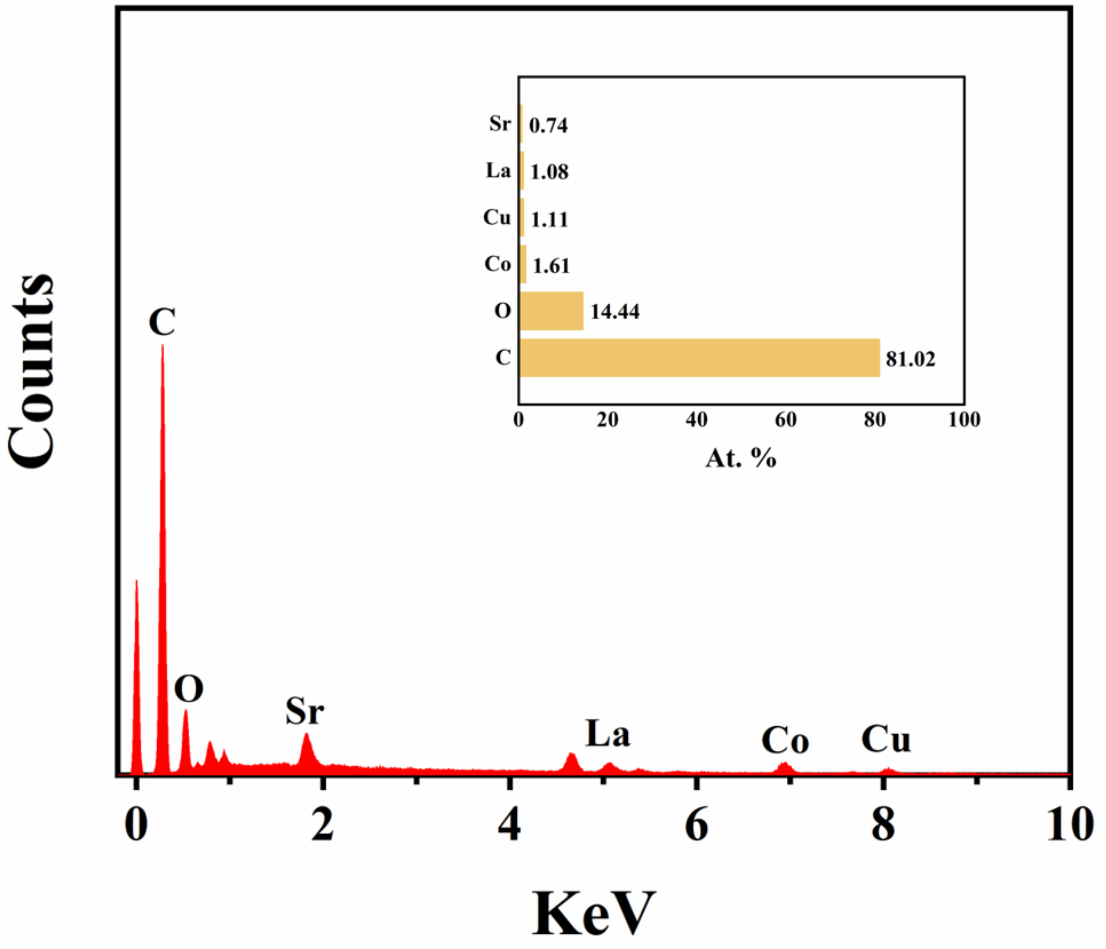

Figure S2. EDX of the LSCC-500 nanofibers.

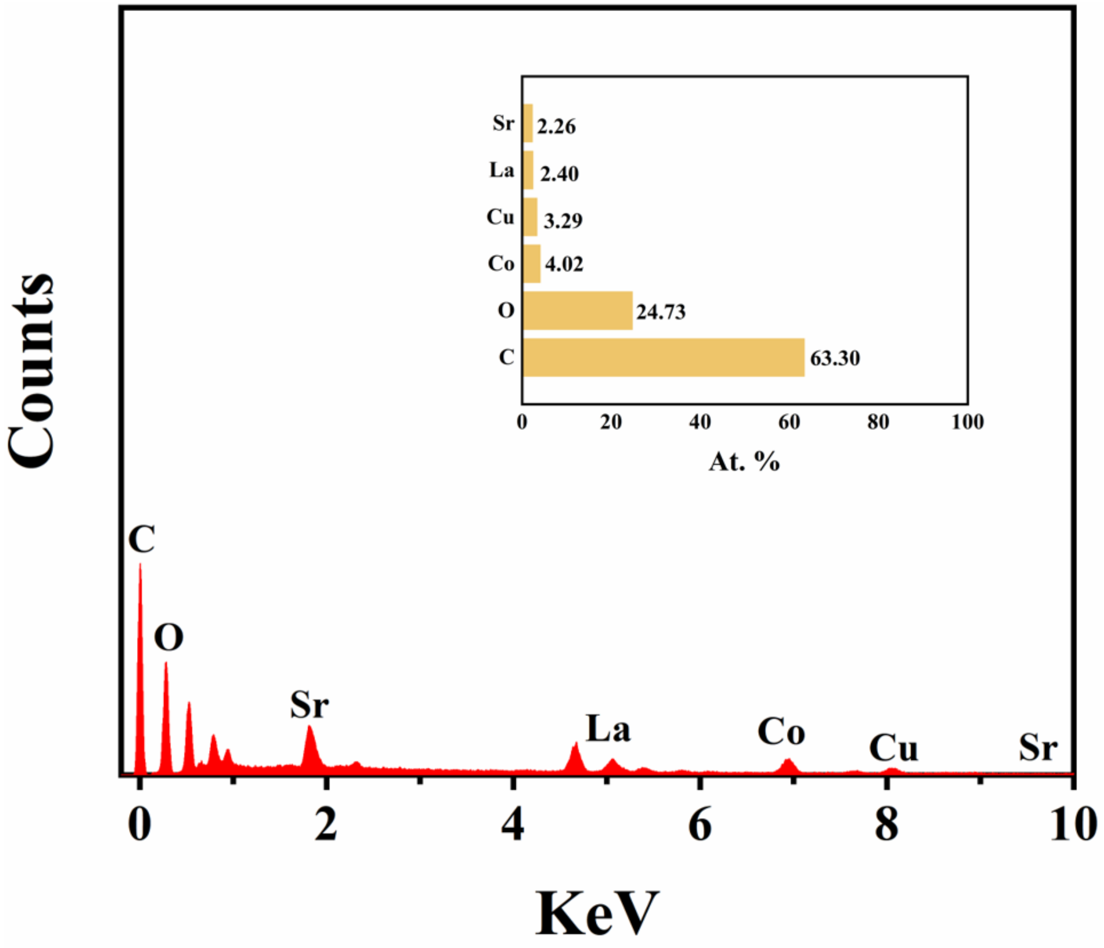

Figure S3. EDX of the LSCC-550 nanofibers.

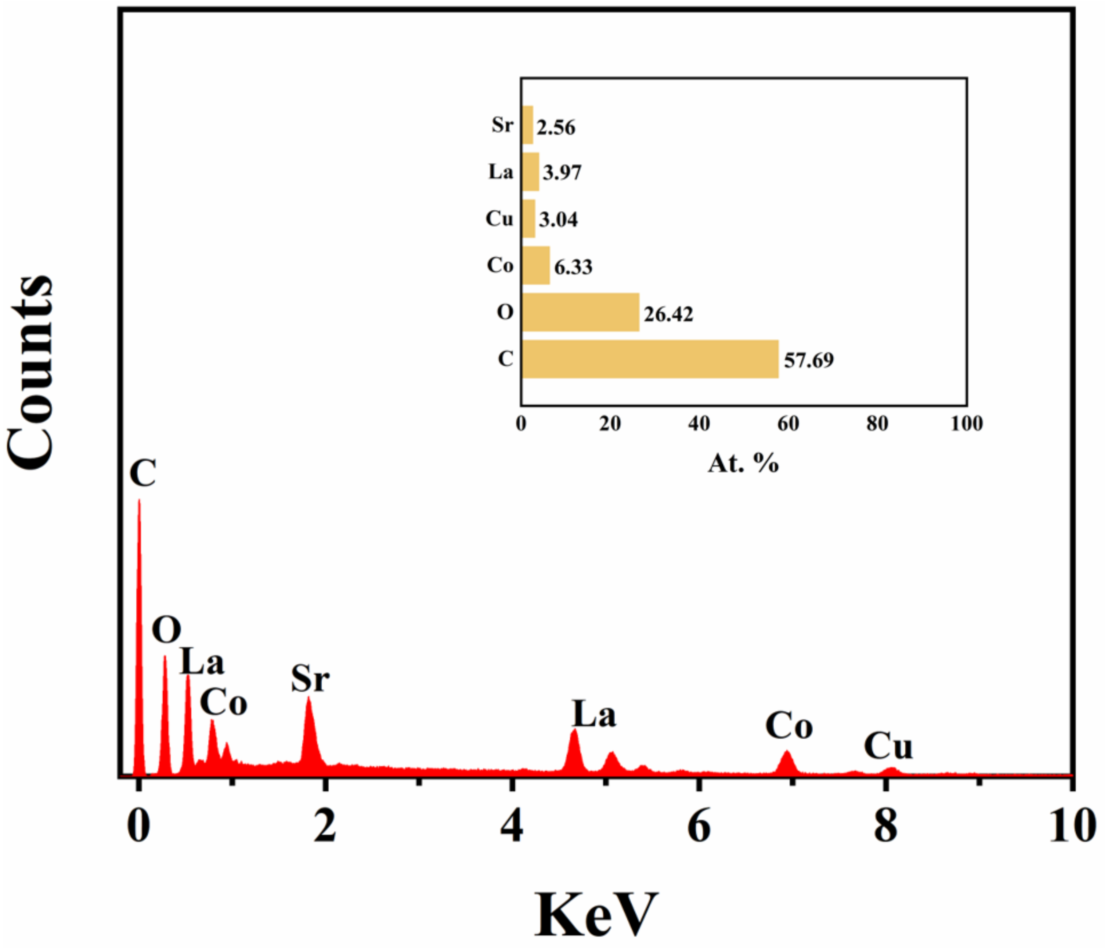

Figure S4. EDX of the LSCC-600 nanofibers.

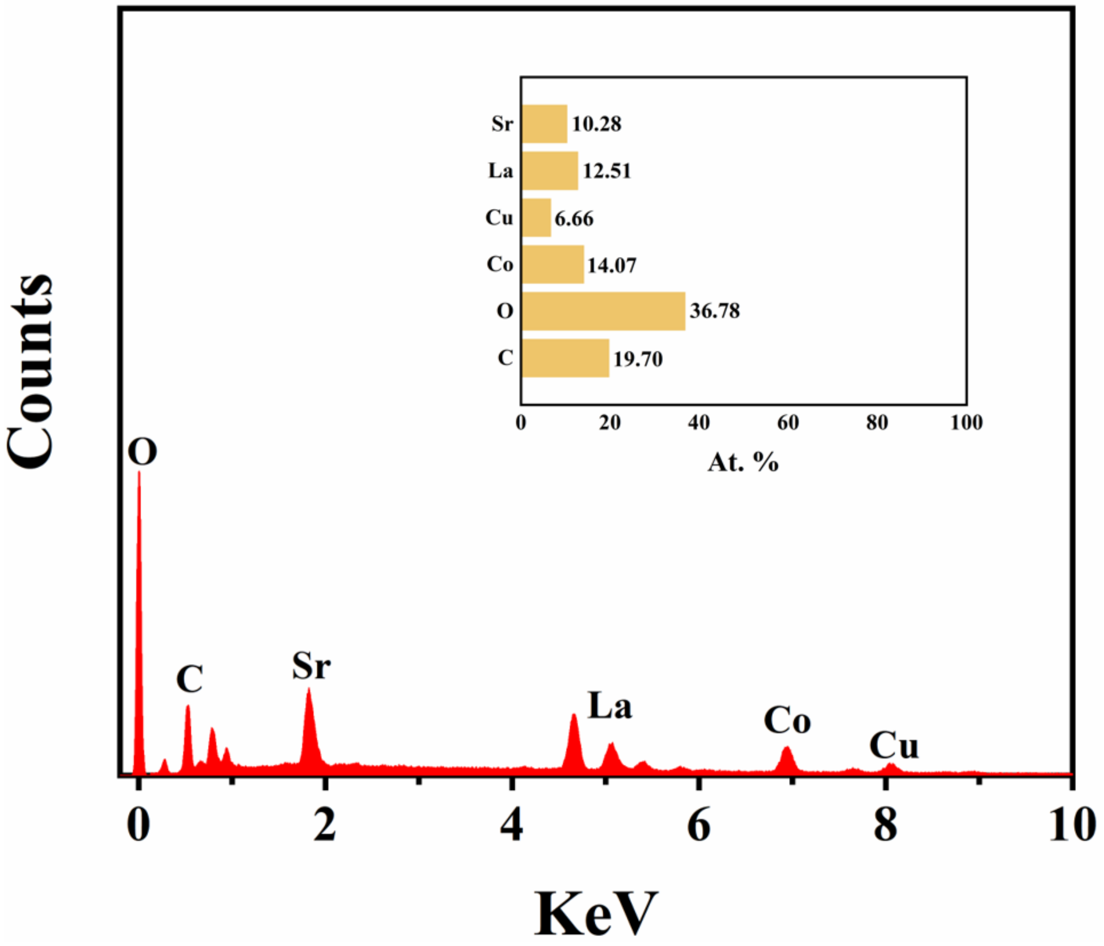

Figure S5. EDX of the LSCC-650 nanofibers.

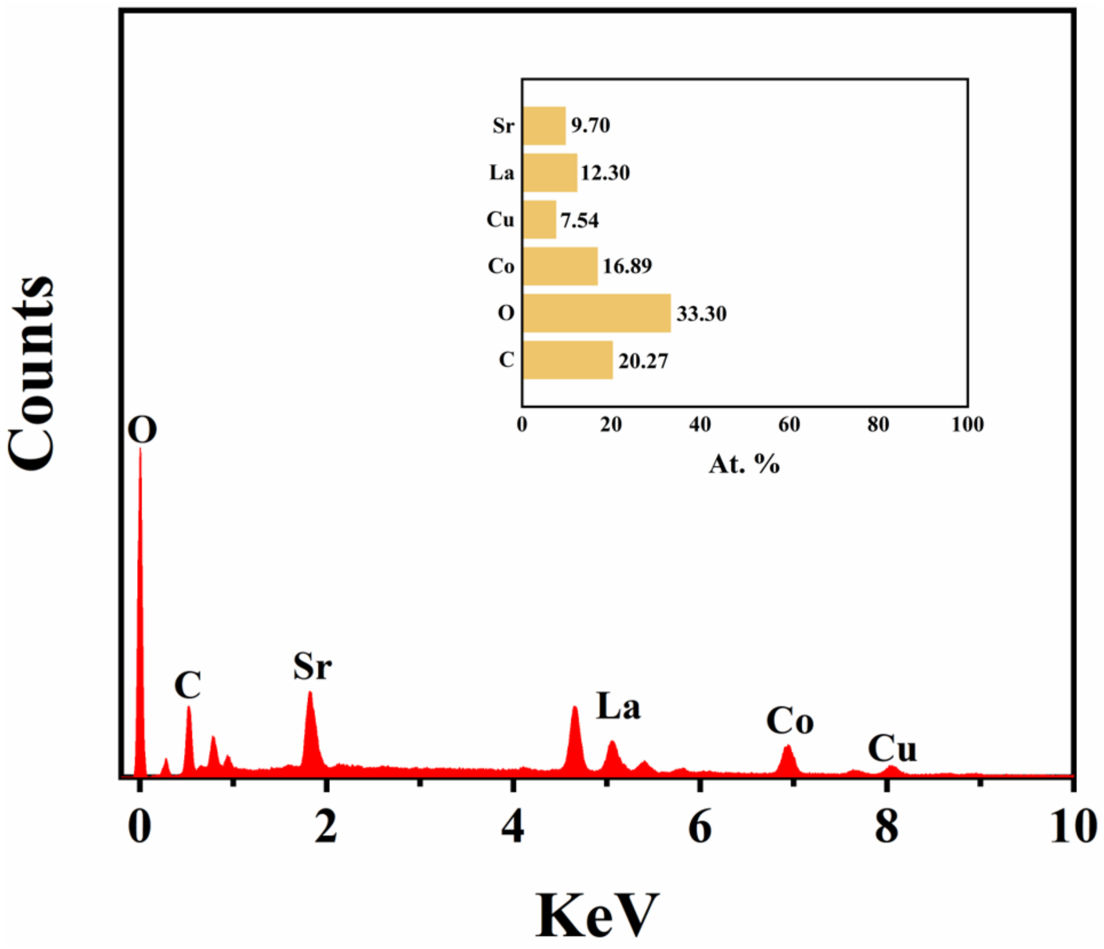

Figure S6. EDX of the LSCC-700 nanofibers.

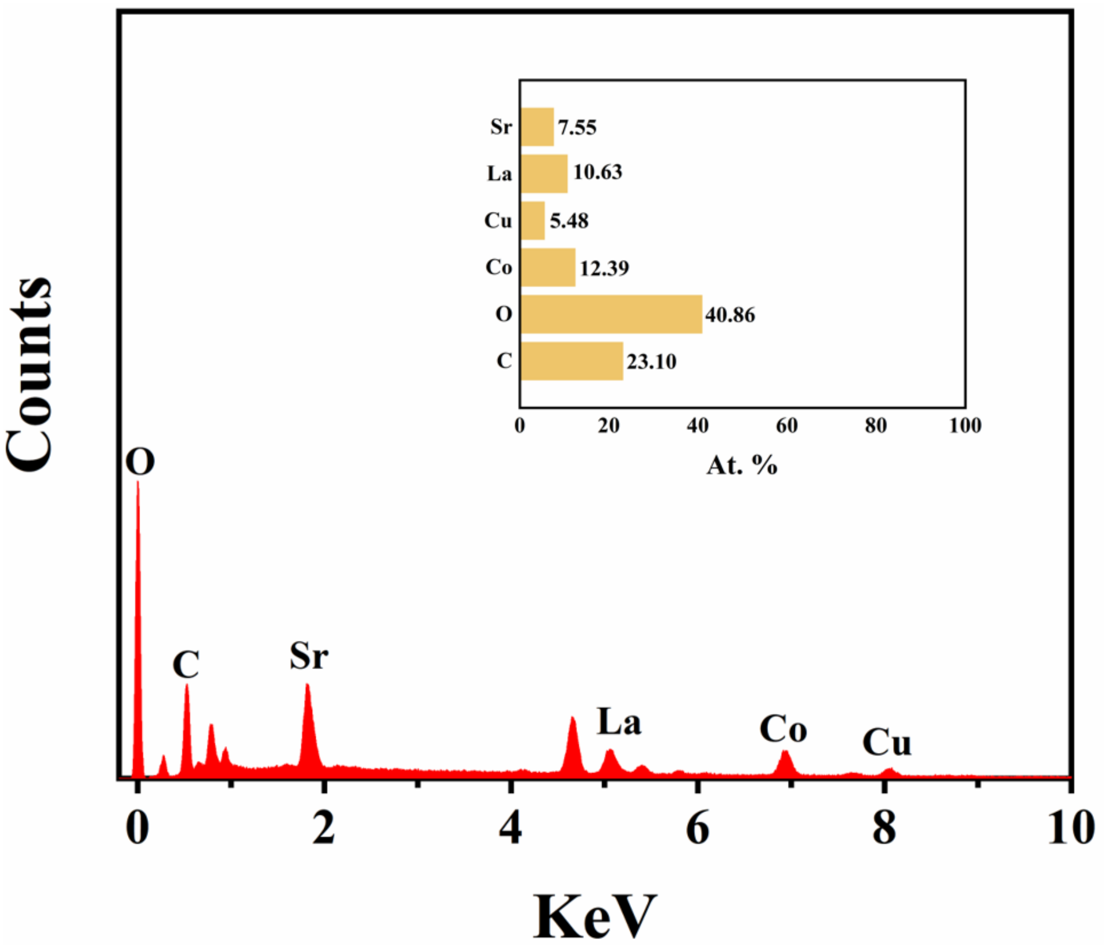

Figure S7. EDX of the LSCC-750 nanofibers.

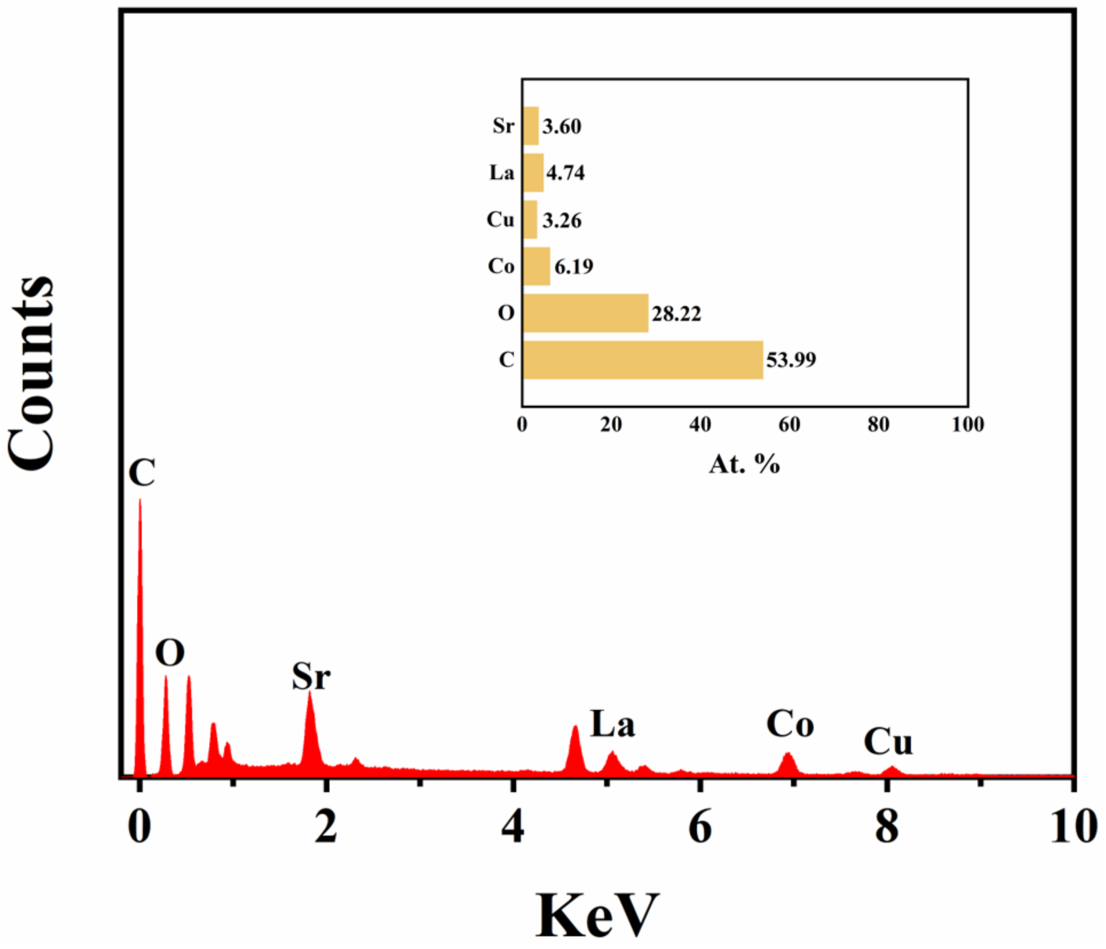

Figure S8. EDX of the LSCC-800 nanofibers.

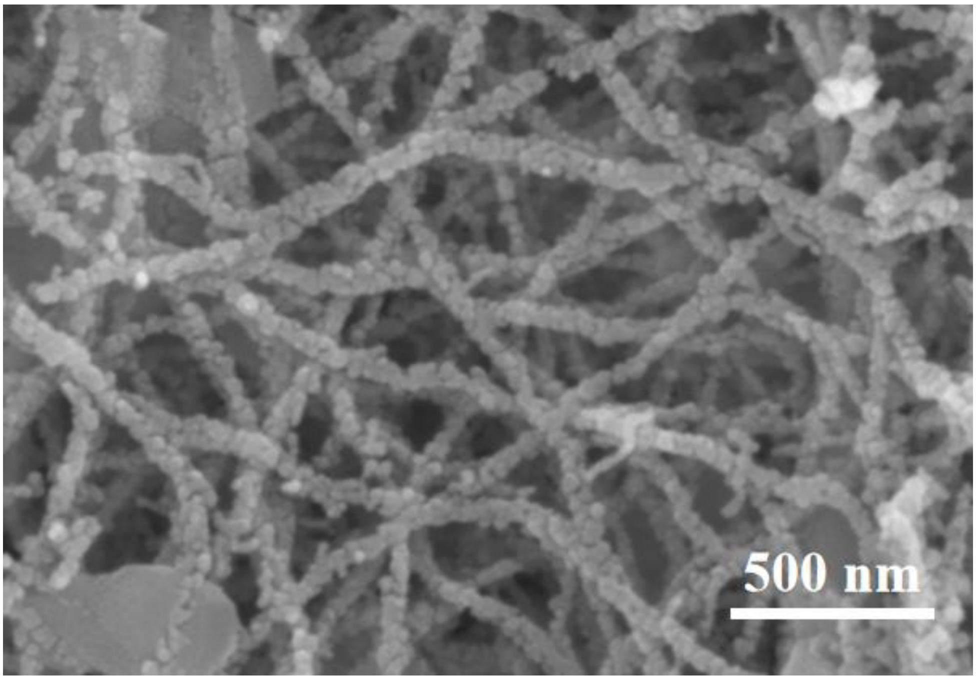

Figure S9. SEM figure after 5000 cycles images of the LSCC-650 nanofibers.

Notes and references

1. Zhang, Z.; Chen, Y.; Li, H.; Hua, H. Simulation and experimental study on vibration and sound radiation control with piezoelectric actuators. *Shock Vib.* **2011**, *18*, 343–354.
2. Li, P.; Wan, X.; Su, J.; Liu, W.; Guo, Y.; Yin, H.; Wang, D. A single-phase FeCoNiMnMo high-entropy alloy oxygen evolution anode working in alkaline solution for over 1000 h. *ACS Catal.* **2022**, *12*, 11667–11674.
3. Liu, H.; Xie, R.; Wang, Q.; Han, J.; Han, Y.; Wang, J.; Fang H, Qi J, Ding M, Ji W, et al. Enhanced OER Performance and Dynamic Transition of Surface Reconstruction in $LaNiO_3$ Thin Films with Nanoparticles Decoration. *Adv. Sci.* **2023**, *10*, 2207128.
4. Li, L.; Jiang, B.; Tang, D.; Zhang, Q.; Zheng, Z. Hydrogen generation by acetic acid steam reforming over Ni-based catalysts derived from $La_{1-x}CexNiO_3$ perovskite. *Int. J. Hydrogen Energy* **2018**, *43*, 6795–6803.
5. Yu, J.; Chen, G.; Sunarso, J.; Zhu, Y.; Ran, R.; Zhu, Z.; Shao, Z. Cobalt oxide and cobalt-graphitic carbon core–shell based catalysts with remarkably high oxygen reduction reaction activity. *Adv. Sci.* **2016**, *3*, 1600060.
6. Alegre, C.; Busacca, C.; Di Blasi, A.; Di Blasi, O.; Aricò, S.; Antonucci, A.V.; Baglio, V. Toward more efficient and stable bifunctional electrocatalysts for oxygen electrodes using $FeCo_2O_4$/carbon nanofiber prepared by electrospinning. *Mater. Today Energy* **2020**, *18*, 100508.
7. Wang, H.; Xu, W.; Richins, S.; Liaw, K.; Yan, L.; Zhou, M.; Luo, H. Polymer-assisted approach to $LaCo_{1-x}Ni_xO_3$ network nanostructures as bifunctional oxygen electrocatalysts. *Electrochim. Acta* **2019**, *296*, 945–953.
8. He, D.; He, G.; Jiang, H.; Chen, Z.; Huang, M. Enhanced durability and activity of the perovskite electrocatalyst $Pr_{0.5}Ba_{0.5}CoO_{3-\delta}$ by Ca doping for the oxygen evolution reaction at room temperature. *Chem. Commun.* **2017**, *53*, 5132–5135.
9. Yi, K.; Sun, L.; Li, Q.; Xia, T.; Huo, L.; Zhao, H.; Li, J., Lü, Z., Bassat, J.M., Rougier, A.; et al. Effect of Nd-deficiency on electrochemical properties of $NdBaCo_2O_{6-\delta}$ cathode for intermediate-temperature solid oxide fuel cells. *Int. J. Hydrogen Energy* **2016**, *41*, 10228–10238.
10. Yu, J.; Wang, Z.; Wang, J.; Zhong, W.; Ju, M.; Cai, R.; Qiu, C.; Long, X.; Yang, S. The role of ceria in a hybrid catalyst toward alkaline water oxidation. *ChemSusChem* **2020**, *13*, 5273–5279.
11. Zhu, P.; Hu, M.; Deng, Y.; Wang, C. One-pot fabrication of a novel agar-polyacrylamide/graphene oxide nanocomposite double network hydrogel with high mechanical properties. *Adv. Eng. Mater.* **2016**, *18*, 1799–1807.
12. Xiao, L.; Liang, Y.; Li, Z.; Wu, S.; Luo, S.; Sun, H.; Zhu, S.; Cui, Z. Amorphous FeNiNbPC nanoprous structure for efficient and stable electrochemical oxygen evolution. *J. Colloid Interface Sci.* **2022**, *608*, 1973–1982.
13. Wang, C.; Wang, R.; Peng, Y.; Chen, J.; Chen, Z.; Yin, H.; Li, J. Nb-incorporated Fe(oxy) hydroxide derived from structural transformation for efficient oxygen evolution electrocatalysis. *J. Mater. Chem. A* **2020**, *8*, 24598–24607.
14. Chen, Q.; Liu, G.; Liu, S.; Su, H.; Wang, Y.; Li, J.; Luo, C. Remodeling the tumor microenvironment with emerging nanotherapeutics. *Trends Pharmacol. Sci.* **2018**, *39*, 59–74.
15. Wang, C.; Cheng, Y.; Ianni, E.; Lin, B. A highly active and stable $La_{0.5}Sr_{0.5}Ni_{0.4}Fe_{0.6}O_{3-\delta}$ perovskite electrocatalyst for oxygen evolution reaction in alkaline media. *Electrochim. Acta* **2017**, *246*, 997–1003.
16. Retuerto, M.; Calle-Vallejo, F.; Pascual, L.; Lumbeeck, G.; Fernandez-Diaz, M.T.; Croft, M.; Gopalakrishnan, J.; Pena, M.A.; Hadermann, J.; Greenblatt, M.; et al. $La_{1.5}Sr_{0.5}NiMn_{0.5}Ru_{0.5}O_6$ double perovskite with enhanced ORR/OER bifunctional catalytic activity. *ACS Appl. Mater. Interfaces* **2019**, *11*, 21454–21464.
17. Kumar, N.; Kumar, M.; Nagaiah, T.; Siruguri, V.; Rayaprol, S.; Yadav, A.; Jha, S.N.; Bhattacharyya, D.; Paul, A.K. Investigation of new B-site-disordered perovskite oxide $CaLaScRuO_{6+\delta}$: An efficient oxygen bifunctional electrocatalyst in a highly alkaline medium. *ACS Appl. Mater. Interfaces* **2020**, *12*, 9190–9200.
18. Sun, Y.; Li, R.; Chen, X.; Wu, J.; Xie, Y.; Wang, X.; Zhang, Y. A-site management prompts the dynamic reconstructed active phase of perovskite oxide OER catalysts. *Advanced Energy Materials*. **2021**, 11, 2003755.

19. Duan, Y.; Yu, Y.; Hu, J.; Zheng, S.; Zhang, T.; Ding, H.; Hu, B.; Fu, Q.; Yu, L.; Zheng, X.; et al. Scaled-up synthesis of amorphous NiFeMo oxides and their rapid surface reconstruction for superior oxygen evolution catalysis. *Angew. Chem. Int. Ed.* **2019**, *58*, 15772–15777.
20. Ning, M.; Zhang, F.; Wu, L.; Xing, X.; Wang, D.; Song, S.; Zhou, Q.; Yu, L.; Bao, J.; Chen, S.; et al. Boosting efficient alkaline fresh water and seawater electrolysis via electrochemical reconstruction. *Energy Environ. Sci.* **2022**, *15*, 3945–3957.
21. Karthik, N.; Atchudan, R.; Edison, T.; Choi, S. Insights of pristine stainless steel mesh oxygen evolution reaction in diverse concentrations of potassium hydroxide. *Mater. Lett.* **2023**, *333*, 133557.
22. Peng, Q.; Zhuang, X.; Wei, L.; Shi, L.; Isimjan, T.; Hou, R.; Yang, X. Niobium-Incorporated $CoSe_2$ Nanothorns with Electronic Structural Alterations for Efficient Alkaline Oxygen Evolution Reaction at High Current Density. *ChemSusChem* **2022**, *15*, e202200827.
23. Alegre, C.; Busacca, C.; Di Blasi, A.; Di Blasi, O.; Aricò, S.; Antonucci, A.V.; Baglio, V. Toward more efficient and stable bifunctional electrocatalysts for oxygen electrodes using $FeCo_2O_4$/carbon nanofiber prepared by electrospinning. *Mater. Today Energy* **2020**, *18*, 100508.
24. Mondal, R.; Ratnawat, H.; Mukherjee, S.; Gupta, A.; Singh, P. Investigation of the role of Sr and development of superior Sr-doped hexagonal $BaCoO_{3-\delta}$ perovskite bifunctional OER/ORR catalysts in alkaline media. *Energy Fuels* **2022**, *36*, 3219–3228.
25. Zhu, C.; Nobuta, A.; Nakatsugawa, I.; Akiyama, T. Solution combustion synthesis of $LaMO_3$ (M= Fe, Co, Mn) perovskite nanoparticles and the measurement of their electrocatalytic properties for air cathode. *Int. J. Hydrogen Energy* **2013**, *38*, 13238–13248.
26. Lu, Y.; Chien, Y.; Liu, C.; You, T.; Hu, C. Active site-engineered bifunctional electrocatalysts of ternary spinel oxides, $M_{0.1}Ni_{0.9}Co_2O_4$ (M: Mn, Fe, Cu, Zn) for the air electrode of rechargeable zinc–air batteries. *J. Mater. Chem. A* **2017**, *5*, 21016–21026.
